# Training with brain-machine interfaces, visuo-tactile feedback and assisted locomotion improves sensorimotor, visceral, and psychological signs in chronic paraplegic patients

**DOI:** 10.1371/journal.pone.0206464

**Published:** 2018-11-29

**Authors:** Solaiman Shokur, Ana R. C. Donati, Debora S. F. Campos, Claudia Gitti, Guillaume Bao, Dora Fischer, Sabrina Almeida, Vania A. S. Braga, Patricia Augusto, Chris Petty, Eduardo J. L. Alho, Mikhail Lebedev, Allen W. Song, Miguel A. L. Nicolelis

**Affiliations:** 1 Neurorehabilitation Laboratory, Associação Alberto Santos Dumont para Apoio à Pesquisa (AASDAP), São Paulo, Brazil; 2 Associação de Assistência à Criança Deficiente (AACD), São Paulo, Brazil; 3 Brain Imaging and Analysis Center, Duke Univ Medical Center, Durham, NC, United States of America; 4 Department of Neurosurgery, University of Sao Paulo Medical School, Sao Paulo, Brazil; 5 Department of Neurobiology, Duke University Medical Center, Durham, NC, United States of America; 6 Duke Center for Neuroengineering, Duke University, Durham, NC, United States of America; 7 Department of Biomedical Engineering, Duke University, Durham, NC, United States of America; 8 Department of Neurology, Duke University, Durham, NC, United States of America; 9 Department of Neurosurgery, Duke University, Durham, NC, United States of America; 10 Department of Psychology and Neuroscience, Duke University, Durham, NC, United States of America; 11 Edmond and Lily Safra International Institute of Neuroscience, Macaíba, Brazil; University of Rome, ITALY

## Abstract

Spinal cord injury (SCI) induces severe deficiencies in sensory-motor and autonomic functions and has a significant negative impact on patients’ quality of life. There is currently no systematic rehabilitation technique assuring recovery of the neurological impairments caused by a complete SCI. Here, we report significant clinical improvement in a group of seven chronic SCI patients (six AIS A, one AIS B) following a 28-month, multi-step protocol that combined training with non-invasive brain-machine interfaces, visuo-tactile feedback and assisted locomotion. All patients recovered significant levels of nociceptive sensation below their original SCI (up to 16 dermatomes, average 11 dermatomes), voluntary motor functions (lower-limbs muscle contractions plus multi-joint movements) and partial sensory function for several modalities (proprioception, tactile, pressure, vibration). Patients also recovered partial intestinal, urinary and sexual functions. By the end of the protocol, all patients had their AIS classification upgraded (six from AIS A to C, one from B to C). These improvements translated into significant changes in the patients’ quality of life as measured by standardized psychological instruments. Reexamination of one patient that discontinued the protocol after 12 months of training showed that the 16-month break resulted in neurological stagnation and no reclassification. We suggest that our neurorehabilitation protocol, based uniquely on non-invasive technology (therefore necessitating no surgical operation), can become a promising therapy for patients diagnosed with severe paraplegia (AIS A, B), even at the chronic phase of their lesion.

## Introduction

Spinal Cord Injuries (SCI) cause a wide array of disabilities with devastating motor, sensory, and autonomic deficits that impair the functional capacity of patients to perform routine living and working activities. SCI also leads to significant impairments in the patient’s quality of life (QoL) [1], his/her body image [2] and sexuality [3]. As such, SCI rehabilitation must consider the patient’s physical, emotional, social and affective life aspects [4], aiming at promoting the patient’s physical independence and autonomy, while promoting the reintegration of the individual into society. Epidemiological studies on SCI [5] reveal a global incidence, considering traumatic or non-traumatic etiology, around 250,000 to 500,000 new cases added every year.

The severity of clinical impairment and recovery prognosis depends on the SCI level [6], the extension and mechanism of injury, presence/absence of residual spinal cord fibers and pre-existing clinical comorbidities. In the US, the most common rank observed one year after the original SCI is AIS A (34%), which includes patients with complete neurological loss SCI (but not necessary anatomically complete lesions [7–9]) [10]. The majority of such AIS A cases involves lesions at the thoracic level (67%) [10]. Studies show a considerable amount of spontaneous improvement during the first year following the lesion [11,12], and stagnation at the chronic phase. For example, following 26 weeks after the original lesion, the rates of motor score recovery for AIS A patients drops to less than 1 point (on a scale where complete paraplegia is 0, and normal function is 50). Between 1 and 5 years after the SCI injury, only about 3.5% of AIS A patients are upgraded to AIS B, 1.05% to C and 1.05% to D [13,14]. These statistics indicate that the most commonly observed cases of chronic paraplegic SCI are represented by AIS A patients who have very little chance of spontaneous neurological recovery at the chronic phase of the injury.

The rehabilitation process with SCI patients involves both learning of tasks as in the use of a wheelchair, and a variety of compensation mechanisms to recover from lost motor functions [15]. The use of stem cell therapy for complete SCI has also been investigated in recent years, both experimentally and clinically, but despite some interesting outcomes, it has not yet been established as a standard effective treatment [16]. Potential new treatments for controlling neuropathic pain, spasticity, bladder, and intestinal functions have also been extensively studied. Those include electrical spinal cord stimulation, chemical neuromodulation [17], drug delivery pumps with catheters inserted in the subarachnoid space [18] and sacral stimulators [19–21].

Despite important recent improvements in rehabilitation techniques, the chances of neurological recovery for motor complete SCIs (AIS A/B) remain low. Indeed, whereas a number of neurorehabilitation protocols have induced some level of neurological recovery in motor incomplete SCI patients (AIS C/D) (including stepping training [22,23], operant conditioning [24,25] or functional electrical stimulation [26]), improvement in motor complete SCI has been principally observed through compensatory mechanisms [15]. Only recently, a few studies in rats [27] and humans [28] have shown partial motor recovery (neurological and functional) in severe cases of SCI, following training with invasive epidural stimulation (see [29] for a review) or invasive pelvic nerve stimulation [30]. Notably, clinical improvement was noticed when such invasive stimulation was paired with direct patient control of the stimulating system, via a brain-machine interface [31] (see [32] for a review).

In a previous study, we have reported significant levels of neurological recovery in eight chronic motor complete (3–13 years post-lesion) SCI patients, after 12 months of training with a multi-step, non-invasive neurorehabilitation protocol [33]. Named the Walk Again Neuro-Rehabilitation (WA-NR), this protocol combines locomotion training, brain-machine interfaces (BMIs [34]) and visuo-tactile feedback. In the WA-NR protocol, SCI patients learn to use their brain activity, recorded via EEG, to control the locomotion of virtual human avatars and robotic gait devices. To close the control loop, continuous streams of tactile feedback are delivered to the skin of patients’ forearms, via a haptic display referred to as the tactile shirt [35], in synchrony with regular visual feedback.

In the present study, we report a detailed analysis of a subgroup of patients from our original study, including six chronic (3–13 years post-lesion) AIS A patients and one AIS B (6 years post-lesion) SCI patient, who continued to train under the WA-NR protocol for a period of 28 months. As a result of this training, we observed significant levels of neurological recovery which included improvements in nociceptive, tactile, and proprioceptive function, a marked improvement in multiple visceral functions (bladder control, bowel function and sexual functions in some patients), and significant gains in voluntary motor control of the lower-limbs (confirmed by both clinical evaluation [6], and neurophysiological measurement (EMGs)). The patients’ anatomical lesion level was revealed by MRI analysis of their SCI. Overall, we observed that the observed sensory and motor improvements occurred in the areas of the body innervated by portions of the spinal cord below the original anatomical SC lesion. As a consequence of such major sensory recovery, we identified a complex reorganization in the patients’ perception of their bodies. Finally, we observed that this partial neurological recovery induced significant improvement in both the psychological and physical aspects of the patients’ self-report on their quality of life [36].

By the end of the training, all seven patients had improved their AIS grade, representing, as far as we can tell, the largest cohort of chronic complete paraplegic patients reported in the literature to exhibit a consistent partial neurological recovery, following a purely non-invasive neurorehabilitation approach.

## Materials and methods

### Participants

Eight paraplegic patients (Table 1), 27–38 years old, with traumatic and chronic SCI (lesion 3–13 years before onset of the training) at thoracic level (T4-T11), participated in the current study: seven patients, from hereon designated as Group 1 (GR1), including six AIS A patients (P1, P3, P4, P5, P6, P8) and one AIS B (P2), followed the WA-NR protocol [37] for a total of 28 months of training. One subject (P7, AIS A) discontinued the training after 12 months and is therefore discussed separately. The participants in the current study are the same that participated in a previous protocol reported by our group [37]. For clarity, the convention used for subject names is the same in both studies.

**Table 1:** Patients’ demography.

Subject	Training period (months)	Sex	Age	AIS^1^	Lesion Level Clinic^2^ Right Left		Lesion Level MRI^3^	Time Since lesion (years)	Etiol^4^	Time since baseline ASIA months^5^
**P1**	28	F	32	A	T10	T11	No data	13	C	142
**P2**	28	M	26	B	T4	T4	T1-T3	6	C	54
**P3**	28	M	32	A	T11	T10	T10-L1	5	O	30
**P4**	28	M	38	A	T8	T8	T7-T9	5	C	40
**P5**	28	M	36	A	T7	T7	T7-T10	3	C	22
**P6**	28	M	29	A	T4	T4	T3-T5	8	C	54
**P7**	12	M	27	A	T5	T7	No data	6	C	62
**P8**	28	F	29	A	T11	T11	T4-L4	9	C	110

1) ASIA neurological standards evaluation

2) Clinical lesion level (AIS)

3) Anatomic lesion level (MRI guided).

4) Traumatic Etiology. C: Closed Trauma; O: Opened Injury.

5) The first measurement is done by the clinical institution that followed them before their enrollment in our protocol done at n months before the onset of our training.

Initially, the level and the grade of each SCI was estimated, using the ASIA standard assessment [6] (Table 1). This measurement was done within the first and the third year post-lesion and is referred to in this paper as the *baseline*. Patients’ original AIS classification and neurological status were confirmed by our medical team at the onset of training (referred to as time 0). Details of patients ASIA score is reported in S1 Fig. To complete our neurological investigation, we examined the patients’ spinal cord using MRI. This measurement was performed once at the end of the protocol. Patient P1 was excluded from the MRI examination for safety reasons (presence of undefined material used for arthrodesis). Data from patient P7 were not recorded due to protocol discontinuation. For three out of the six tested patients, namely P2, P3, and P4, MRI images revealed the existence of some degree of spinal cord continuity at the lesion level (S2 Fig and S1 Video). MRI analysis was partially compromised by artifacts due to the presence of metallic implants in two cases (P5, P6) and by the complexity of the injury in one case (P8), making it difficult to distinguish between neurological and pathological tissue.

To assess the effect of our intervention, we clinically evaluated GR1 patients eight times: at the protocol onset, and after 4, 7, 10, 12, 16, 22 and 28 months of training. Patient P7 was evaluated during the first year and then once at the end of 28 months, after 16 months of training discontinuation.

### Inclusion criteria

Subjects were adult paraplegics, grade AIS A (complete), B (motor complete) (3) with traumatic SCI at the thoracic level, at least 6 months before the onset of the study, with the absence or offset comorbidities, and emotionally stable. We excluded patients with non-traumatic SCI, decompensated comorbidities, a degree of spasticity exceeding a score of 2 (on the Ashworth scale), degree of osteoporosis (T- score) < -4, and presence of joint deformities, fractures, pressure ulcers grade > 3, peripheral neuropathy of the upper limbs, brain injury, degenerative neuromuscular injury amputation of upper or lower limbs, pacemakers (cardiac/neural), cephalic (cranial/brain) implants and with emotional instability.

### Design of experiment

The WA-NR protocol [33] consisted of two main classes of training: active locomotion training (TR-LOC) and BMI-based neurorehabilitation exercises (TR-BMI). Progressively more complex stages, as part of a multistep strategy, were employed during application of the WA-NR protocol [33], assuring that patients had enough time to acquire upper limb and trunk strengthening, cardiovascular stability, as well as emotional adaption to the experience of orthostatic posture. The TR-LOC included training with both a robotic gait training device (Lokomat) and a body weight support system (ZeroG).

The steps for the TR-BMI training included BMI control of a virtual avatar while the patient was in a seated position, BMI control of an avatar while patient was in an orthostatic position, BMI control of the Lokomat, and BMI control of an exoskeleton (all steps of this training are detailed in [33]). Table 2 shows the average number of training sessions for each patient. On average, during the first four months, patients had two interventions per week and once per week for the rest of the protocol. The period 10–12 corresponded to a break period for all the patients, with no training involved.

**Table 2:** Average monthly training.

Periods (Months range)		0–4	4–7	7–10	10–12	12–16	16–22	22–28
**TR-BMI**	P1	4	3	1	0	1	1	0
	P2	5	4	2	0	0	2	0
	P3	5	5	2	0	2	2	0
	P4	5	5	1	0	1	1	0
	P5	6	4	1	0	1	2	0
	P6	5	4	1	0	0	1	0
	P7	3	5	1	0	0	0	0
	P8	3	2	1	0	1	1	0
**TR-LOC**	P1	3	0	2	0	2	1	1
	P2	2	0	2	0	2	2	1
	P3	2	0	3	0	2	2	1
	P4	3	0	3	0	2	2	2
	P5	4	0	2	0	2	2	1
	P6	2	0	3	0	1	2	2
	P7	3	0	2	0	0	0	0
	P8	2	0	2	0	1	1	1

Values are average numbers of sessions per month for BMI-based neurorehabilitation exercises (TR-BMI) and active locomotion training (TR-LOC).

#### Active locomotion training

TR-LOC activities included locomotion training with a body weight support (BWS) system, robotic gait therapy device on a treadmill (Lokomat, Hocoma), and training with an over-ground fixed track BWS system (ZeroG, Aretech LLC). During gait training, subjects were guided to attempt to execute lower-limb motor tasks actively, despite the SCI. For the Lokomat training, the physiotherapist verbally instructed the patients to try to actively perform lower limb movements, in conjunction with the computer-generated orthosis movements, which included flexion and extension of hip, knee, and ankle. Each session was distributed in blocks, during which specific movements were trained; for instance, hip flexion during swing phase. The device provided real-time biofeedback regarding the patient’s joints torque. BWS was limited by the maximum knee extension, without joint collapse during the stance phase of gait (up to 80% of BWS, avoiding further reductions in this device). Guidance force was fixed at 100%, and treadmill speed was set between 1–1.5 km/h, to promote the safest possible training environment. During ZeroG training, subjects wore leg orthosis (for joint stabilization: hip, knee, ankle), and employed a wheeled triangular walker, while being assisted by the physiotherapist. BWS was progressively decreased from 75% to 30% throughout the sessions (bone densitometry guided the lower BWS level). The training promoted postural control, dynamic balance control, cardiovascular conditioning, upper limb and trunk strengthening, lower limb voluntary activation during stance and swing gait sub-phases.

#### BMI-based neurorehabilitation exercises

**Fig 1:**
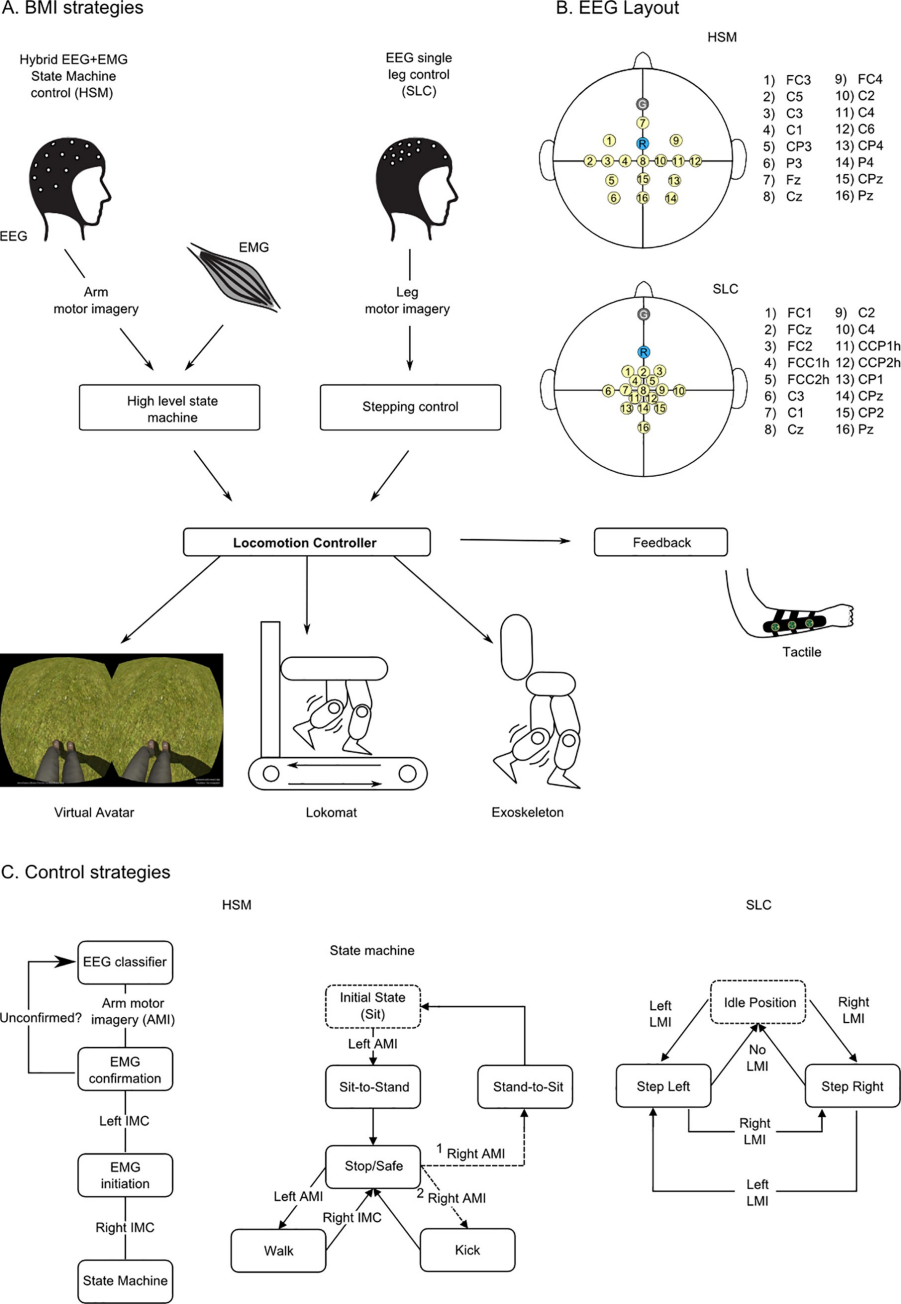
Brain-machine interfaces control strategy. (**A**) We have developed and validated two BMI strategies: the hybrid EEG and EMG state machine control (HSM) and the EEG single leg control (SLC). Both strategies were used to control the actuation of a virtual avatar and a robotic gait device (Lokomat or exoskeleton). A portable haptic device was used to inform the user of the position of the virtual or robotic leg and the contact of these actuator’s feet with the floor in real time [35]. (**B**) For both BMI strategies, a 16 channel EEG cap was used. Electrodes were clustered over the arm area of the sensorimotor cortex for HSM and leg area for the SLC strategy. The ground and reference electrodes are reported in gray and light blue respectively. (**C**) The control strategy for the HSM (left panel) is based on navigation of a state machine (middle panel), using motor imagery, and a two-step EMG confirmation, using isometric muscle contraction of the biceps (IMC). For example, when the subject is in a standing position (Stop/Safe sate) and wants to start walking, (s)he will imagine moving the left arm and confirms the choice with a left bicep IMC. The subject has then to produce a right bicep ICM to trigger walking. The SLC strategy uses the decoding of leg motor imagery through EEG signals. If left motor imagery (LMI) is detected the left step is triggered. Once in this position, if right motor imagery is detected, the right step is triggered, and if no state is detected for 5 seconds, the actuator (the avatar or the robotic gait device) returns to the idle position.

A 16-channel EEG cap was used for all the TR-BMI sessions. Overall, we implemented and tested two BMI control strategies. The hybrid state machine (Fig 1A) strategy employed 16 channel EEG recordings over the arm motor cortex area (Fig 1B) and two channel EMG recordings of the subject's upper arms. Subjects used left and right arm motor imagery to select specific actions in a state machine (Fig 1C) and two stage EMG activation to confirm the selection and the state transition to trigger the action. This two-step confirmation strategy ensured that the movement of the avatar or the robotic legs was not triggered by mistake in case a false positive was detected at the EEG decoding phase. Patients could use this strategy to control sit/stand-up and walk/stop states. Alternatively, the same approach was used for patients to trigger walk/stop and kick states.

The single leg control (SLC) protocol employed 16 channel EEG recordings, clustered around the medial longitudinal fissure, meaning that it was more densely focused over the putative leg motor representation area of the primary sensorimotor cortex (Fig 1B). Subjects used left or right leg motor imagery to trigger the stepping of the corresponding limb.

Both BMI strategies were tested to control a 3D virtual avatar and a robotic leg actuator (Lokomat or a custom-built exoskeleton [38]). An array of vibrotactile actuators placed on the patients’ forearms provided online artificial proprioceptor/tactile feedback regarding the position of the legs (virtual or robotic) during the locomotion and the contact of the robotic actuator feet with the floor. This feedback was delivered to the patients’ forearms using a portable haptic display developed by our team and called the “tactile shirt” [35]. An LED display, integrated into the Lokomat and the exoskeleton, informed the patient about the status of the experiment and the outcome of the EEG classifier.

For both BMI strategies, we used linear discriminant analysis (LDA), using features extracted by a six-dimensional common spatial pattern (CSP) to construct an EEG classifier. The first BMI strategy was tested during the first 6 months of training and the second one for the rest of the protocol.

### Statistics

Given the small samples of subjects, when possible, the details per subject are shown rather than a group average. Graphs with group scores report group mean and SEM.

#### Correlation analysis

To study the main factors that influenced the observed neurological improvement in our patients, we performed a correlation analysis between the improvement rate and training related factors (TR) and external factors (EX). The improvement rate was calculated as the difference of the AIS score (motor or sensory) between two consecutive AIS assessments. We considered the eight measurements done by our team for patients in GR1 (at the onset of training and after 4,7,10, 12, 16, 22 and 28 months) and the six measurements for patient P7.

The training-related factors were: the number of training hours of locomotion training (TR-LOC), and the number of hours with the BMI-based training (TR-BMI) (Table 2). The external factors were the SCI height (EX-SCH, listed from cervical to sacral, i.e., first cervical C2 = 1 and last sacral dermatome S4-S5 = 28), the time since the lesion (EX-TME), and the patients’ age (EX-AGE). For the correlation analysis between the improvement rates and the experiment factors, the Pearson correlation coefficient and p-values of the correlation are calculated with the standard corrcoef Matlab function.

### Clinical assessments

#### Magnetic resonance imaging (MRI)

Spinal cord MRI scans, using a 1.5T GE-Genesis equipment, with gadolinium-based intravenous contrast, were collected 28 months after the training onset. Images were obtained in axial, sagittal and coronal planes, and in T1, T2 and FIESTA (Fast Imaging Employing Steady-state Acquisition) sequences. Submillimeter cuts were evaluated by a radiologist blinded to the experimental paradigm to detect possible residual neural fibers at the level of the SCI. An additional 3D reconstruction of the spinal cord was obtained using the following method: (1) a spinal cord toolbox [39] was used to align the slices of the spinal cord; (2) centerline image was set manually on each axial slice, and (3) segmentation was obtained with sct_propseg function within the cerebrospinal fluid and confirmed by an experienced doctor. The three-dimensional mesh was rendered with ITK-SNAP software.

#### Neurological evaluation: Sensory and motor scores

A somatosensory score was calculated by summing left and right dermatomes with normal (coefficient 2) and altered (coefficient 1) sensation for each patient [6]. Then, the total somatosensory improvement was calculated as the difference between the score after n months of training and the initial score. The motor evaluation followed the standard AIS motor assessment methodology [6]. Motor evaluation was conducted through a functional examination of 12 muscles, among them five key muscles (rectus femoris proximal portion, rectus femoris distal portion, tibialis anterior, extensor hallucis longus, gastrocnemius) and seven non-key muscles (hip adductors, gluteus maximus, gluteus medius, medial and lateral hamstring,flexor hallucis longus and extensor digitorum longus). Note that the presence of voluntary anal contraction and/or presence of any motor function (grade 1 or above) more than three levels below the motor level on a given side will determine if a patient is AIS B or AIS C (6). The motor score described the level of voluntary strength below the SCI level for each patient, ranging from 0 (absence of contraction) to 5 (for normal contraction, produced against gravity and strong opposing force). The lower extremity motor score (LEMS) was obtained by summing all key muscles (score for a healthy subject is 50, i.e., five key muscles, with a maximal score of 5, bilaterally).

#### Proprioception measurements

For the proprioception evaluation, the experimenter performed individualized joint mobilizations at the lower limbs: hip flexion and extension (F/E), knee F/E, ankle dorsiflexion/plantarflexion, hallux F/E and toes (2^nd^ to fifth) F/E. The patient was blindfolded and in a horizontal supine position, while the examiner performed manual joint mobilization at an approximately angular speed of 60°/second. For the hip flexion, we lifted the leg up maintaining the knee extended and the lower limb aligned, to exclusively move the hip towards flexion, up to 60° (initial position = 0°). For the hip extension, the initial position is 60° of flexion, lowering the leg towards the clinical table. To perform knee mobilizations, we kept the femur lifted with a hip flexion of 30°. For knee flexion, we moved the tibia from extension position 0° towards the clinical table, performing 30° of knee flexion. For knee extension, we moved the tibia from 30° of flexion, back to complete extension 0°. For ankle dorsiflexion, we moved the foot up from neutral position (0°) to 10° of dorsiflexion. For ankle plantarflexion, we moved the foot down from the neutral position (0°) to 40° of plantarflexion. For hallux flexion, we moved it down from the neutral position to 45° of flexion. For hallux extension, we moved it up from the neutral position to 45° of extension. For toes flexion, we moved them down from the neutral position to 30° of flexion and finally for toes extension; we moved it up from the neutral position to 40° of extension.

Patients were instructed to describe when (s)he could perceive the stimulus. We performed 10 different mobilizations for each leg. Then, we calculated the proprioception score as the number of joints in which mobilization was perceived, meaning that a maximum score of 20 corresponded to a subject with proprioceptive function present at the entire lower limb area.

#### Vibration measurement

For this assessment, a vibrating diapason was placed on different bony prominences, including: the ribs; anterior superior iliac spine (hip); patella (knee); medial and lateral malleolus (ankle); hallux, calcaneus bone and the sole of the foot (foot), while patients remained blindfolded in a supine position. Patients described the vibration sensations they experienced after the stimuli were delivered to the trunk and later to the lower limbs, following a random sequence that included bilateral areas like the anterior superior iliac spine, patella, medial and lateral malleolus, calcaneus, hallux, and the sole. Patients were asked whether they could feel the vibration and to describe the stimulus, considering two parameters: location (which area) and side of the body (right or left). The vibration score for each patient was obtained by summing the regions (rib, hip, knee, ankle, and foot) where the patient confirmed perceiving the stimulation (independently from their ability to place it on the correct body part or not). Note that the vibration and the confusion map should be considered as a whole to describe each patient’s vibration perception, the first one indicating if the subject can detect the presence of the stimulus, and the second describing the ability to localize it properly. To avoid a mechanism of learning by association, no feedback on the outcome was given to the patient; the experimenter did not tell the patient if his/her answers were correct or not. Also, throughout the assessment, the patients stayed blindfolded and in a supine position.

#### Autonomic Visceral function evaluation

Visceral recovery was measured using questionnaires that were based on ISCOS datasets for urinary, intestinal and sexual functions [40–43], in addition to direct clinical measurements included in the AIS sacral exam to evaluate anal sphincter function [6].

#### EMG recording and analysis

EMG activity was recorded using bipolar surface electrodes, amplified with actiCHamp amplifier (Brain Vision LLC, Morrisville, NC), and digitized at 2000HZ. Openvibe, an open-source software, was used for data collection [44]. A linear EMG envelope was obtained by rectifying and low-pass filtering the EMG signal, using the 2^nd^ order anticausal Butterworth with 1Hz cutoff frequency. During the test, patients were suspended by the body weight support (Hocoma AG, Switzerland) while their legs were attached to the Lokomat working in passive mode (motors off and backdrivable). The physiotherapist verbally instructed patients to flex and extend the left or right hip for 4 seconds and to relax in between tasks.

#### Quality of life assessment WHOQOL-BREF

The WHOQOL-BREF [36] is a cross-culturally valid self-assessment questionnaire with four Quality of life (QoL) domains: physical, psychological, social relationships and environment (see S1 Table). The score ranges from 1 to 5, on a Likert scale, according to the graduation of agreement or disagreement of the participant. The scores pointing 0 indicate poor QoL and 100 indicate good QoL. Note that higher values in WHOQOL-BREF always mean a positive effect; as such, a high score for the question ‘How often do you have negative feelings such as blue mood, despair, anxiety, depression?’ means low occurrences of negative feelings in the patient.

### Study approval

Our protocol was approved by both the local ethics committee (Associação de Assistência à Criança Deficiente, Sao Paulo, Sao Paulo, Brazil #364.027) and the Brazilian Federal Government Ethics Committee (CONEP, CAAE: 13165913.1.0000.0085). All participants signed written informed consent before enrolling in the study.

## Results

### Sensory improvement

**Fig 2:**
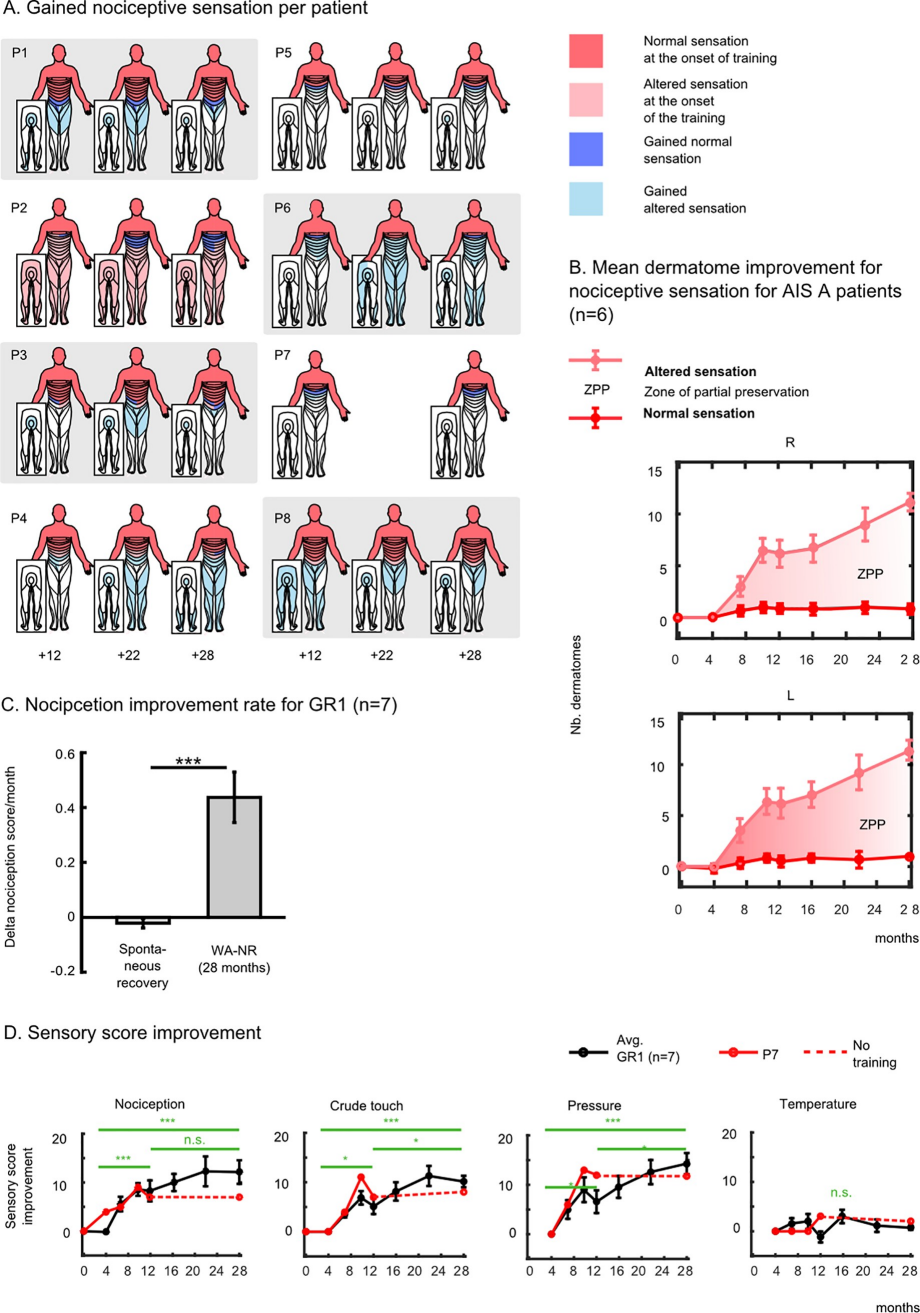
Sensory improvement following the WA-NR training protocol. (**A**) Patients’ normal (dark pink) and altered nociceptive sensations (light pink) areas measured with the standard ASIA assessment [6] at the onset of the training. Dermatomes in dark blue represent body areas where patients recovered normal sensations after our training; areas in the light blue are those where patients gained altered sensation (reported after 12, 22 and 28 months of training). (**B**) Mean ± standard error of the mean (SEM) for gained dermatomes with normal (dark pink), altered (light pink) and zone of partial preservation (ZPP) with nociceptive sensation for GR1 patients except P2. (**C**) Mean±SEM of the nociception improvement rate during the period of the WA-NR (difference of score between the onset and the end of the training, normalized by the number of months between the two evaluations) compared to the mean improvement rate before the training (difference of score between baseline and onset measurement normalized by the number of months between the two measurements). (**D**) Means ± SEM for the gained sensory score for each assessment of GR1 patients for nociceptive sensation, crude touch, sensitivity to pressure and temperature compared to the onset of training. Patient P7 stopped the training after 12 months and was therefore reported separately from the group average (in red). The score obtained after 4 months of training is compared to the one registered after 12 and 28 months of training (t-test, * P<0.05, ** P<0.01, *** P<0.001).

Fig 2A depicts the normal tactile (dark pink) and altered (light pink) sensitivity (hyper or hypo-sensitivity)–which defines the zone of partial preservation (ZPP)–exhibited by each patient at the onset of the training. In this figure, each patient’s gains regarding normal (light pink) and altered (light blue) somatic sensitivity are also plotted for 12, 22 and 28 months of training. Overall, five patients (P1, P3, P4, P6, P8), all diagnosed with complete paraplegia (AIS A) at the onset of the training, recovered nociception in their trunk and lower limbs. The most dramatic improvement was observed in patient P6, whose T4 SCI occurred 8 years prior to our study. At the onset of training, this patient’s ZPP extended to T5-T6. However, 22 months after training onset, P6’s ZPP increased by 15 dermatomes on the right side, and 16 dermatomes on the left side. Patient P2 (T4, AIS B, motor complete) recovered normal nociceptive sensation in three to six dermatomes below the original SCI. Patient P5 regained altered sensations in the sacral dermatomes (S4-S5) 28 months after the training onset, even though he did not exhibit sensitivity below T7 on both right and left sides at the onset of training.

The average increase of ZPP for the GR1 patients (excluding P2 and therefore only AIS A patients) is shown in Fig 2B. On average, patients recovered partial nociceptive sensation in areas situated 11 dermatomes below their original lesion (11.17 on the right side and 11.33 on the left side). Overall, the patients’s sensory score improvement rate for nociception during the 28-month WA-NR period was significantly higher than the spontaneous recovery rate registered before the onset of the training (calculated as the difference between the score registered at onset of the training and the baseline score) (Fig 2C, Wilcoxon rank sum test, n = 7, p<0.001).

Fig 2D displays the temporal evolution of the sensory score improvement for GR1 patients (black line), compared to the score at the onset of the training for nociception (NC), crude touch (CT), pressure (PR) and temperature (TE). The GR1 patients exhibited a consistent and significant increase between the 4^th^ and 28^th^ month of training in NC (12.15 ± 2.42; mean± SE, Wilcoxon test, p<0.001), CT (10.14±1.16, p<0.001), PR (14.28±2.13, p<0.001), but no significant improvement in TE (0.71±0.44, p>0.1).

Periods with reduced training (between the 10^th^ and 12^th^ months) also coincided with the absence of changes (NC) or reduction (CT, PR) in the group averages. Consistent with this observation, patient P7’s scores (continuous red line) stagnated between the 12^th^ and 28^th^ months. Note that during the first year, this patient had exhibited an improvement rate above the GR1 average in NC, CT and PR sensations.

### Proprioception, vibration sensitivity recovery, and body schema

**Fig 3:**
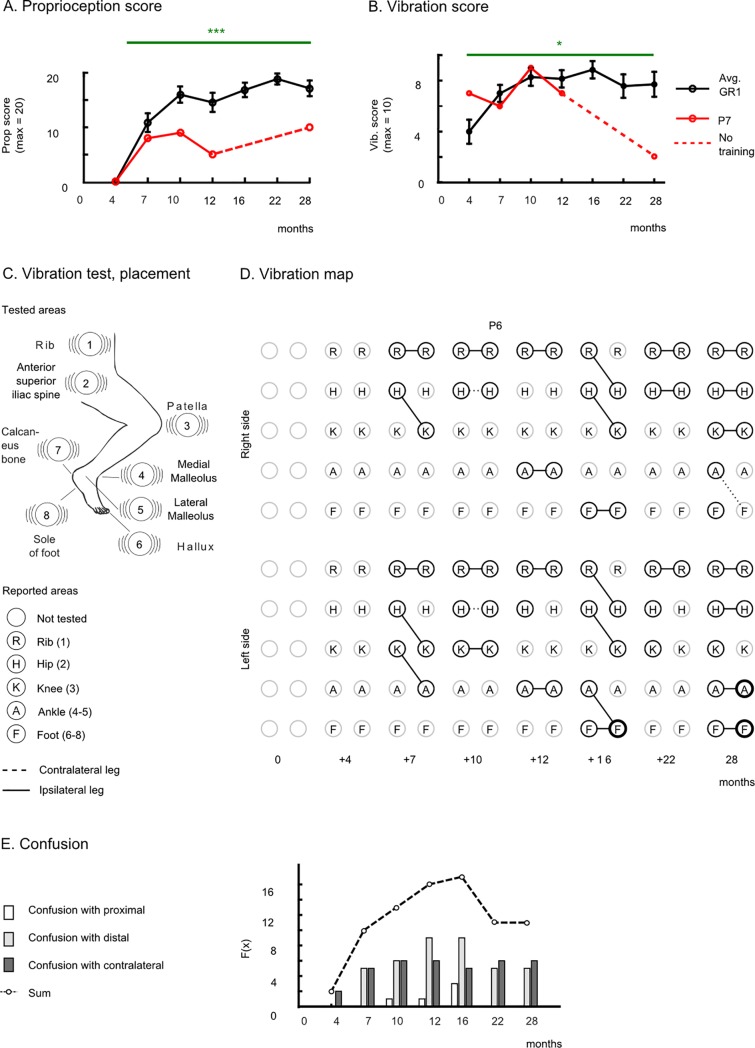
Proprioception and sensitivity to vibration. (**A**) Mean±SEM for proprioception (**B**) for vibration score for GR1 (black) and patient P7 (red). (**C**) Eight stimulation areas for the vibration test. Patients were blindfolded during the exam and had to report if they could feel the vibration and report the location of the stimulation (rib, hip, knee, ankle or foot). (**D**) Vibration map for patient P6. A solid line connects the actual vibration position (left circle (R)ib, (H)ip, (K)nee, (A)nkle and (F)oot) and the felt vibration position (right circle) by the patient. A dashed line means that the patient indicated the contralateral leg. (**E**) Occurrences of confusion toward a proximal joint (e.g., stimulation was done in the knee and patient-reported he felt in the hip), distal joint or with a contralateral joint.

All patients exhibited a significant recovery in lower-limb proprioceptive sensitivity (Fig 3A). This assessment was first introduced 4 months into the training protocol. The mean proprioception score at the end of the 28 months of training was 17.14±1.89 (mean ± SE) over 20, in complete contrast with the measurement at the 4^th^ month of the training, where no patient was able to report proprioceptive sensation in any of the tested joints (average group score = 0.0). After 28 months of training, six out of seven patients recovered proprioception up to the ankle and five patients up to the toes (see S3 Table). Patient P7’s proprioception improved up to the ankle after 10 months of training and 18 months later, despite no further training, this patient still preserved this improvement (Fig 3A).

In parallel, the patients’ vibration sensitivity was enhanced markedly between the first measurement done after 4 months of training (4.0±0.94, mean ±SE) and the 28^th^ month of training (7.71±0.97) (p<0.05, Wilcoxon test) (Fig 2B). Different from the other sensory modalities, we observed a notable decrease in patient P7’s vibration score after protocol discontinuation.

As part of our evaluation protocol, patients were asked to report either the presence or the absence of sensation, and to identify the location on the body where they felt the vibration (Fig 3C and 3D). As training progressed, patients started to recover vibration sensations in more distal parts of the body. However, the newly recovered sensory areas were disorganized in terms of the patients’ body representation. Because of that effect, patients tended to perceive the vibration stimuli on more distal body areas than the point upon which the stimuli were delivered. In Fig 2D we use a new graphical representation, which we refer to as the vibration map, to depict the evolution of patient P6’s recovery on vibration sensation. During the first evaluation (4^th^ month) this patient reported no sensation in both left and right legs. Two months later, the patient started perceiving two regions on the right side (the ribs and the hip) and three on the left side (ribs to the knee). However, when asked to report the stimuli location, the patient reported feeling that the vibration applied to his hips felt like it originated in the knees, whereas the vibration on his left knee felt as if it was delivered to his left ankle. In other words, the patient perceived the stimulation more distal than the actual location to which it was applied. Several other instances of spatial localization errors were observed with this patient throughout the assessments. However, with training, the overall vibration map became more organized, and by the end of the protocol, this patient was able to correctly perceive vibration stimulation up to the knee on the right side and up to the foot on the left side.

Other patients in GR1 also experienced similar errors related to the correct spatial localization of a vibratory stimulus. They included, in addition to locating the stimulus in a more distal joint, locating it in a more proximal joint or in a contralateral joint. In all cases, these errors were triggered by the expansion of the sensory recovery (Fig 3E) below the original SCI. As a rule, patients often located the vibratory stimulus to more distal than proximal joints (39 cases vs. 5 respectively). The instances of misallocating the stimulus to a contralateral joint stayed constant over the training period (overall 36 cases). The highest incidence of these spatial localization errors took place between the 12^th^ and the 16^th^ month of training. After 22 months of training, and following a period in which the vibration score plateaued, the number of errors decreased. Consequently, the resulting vibration map became more organized.

### Motor function improvements

**Fig 4:**
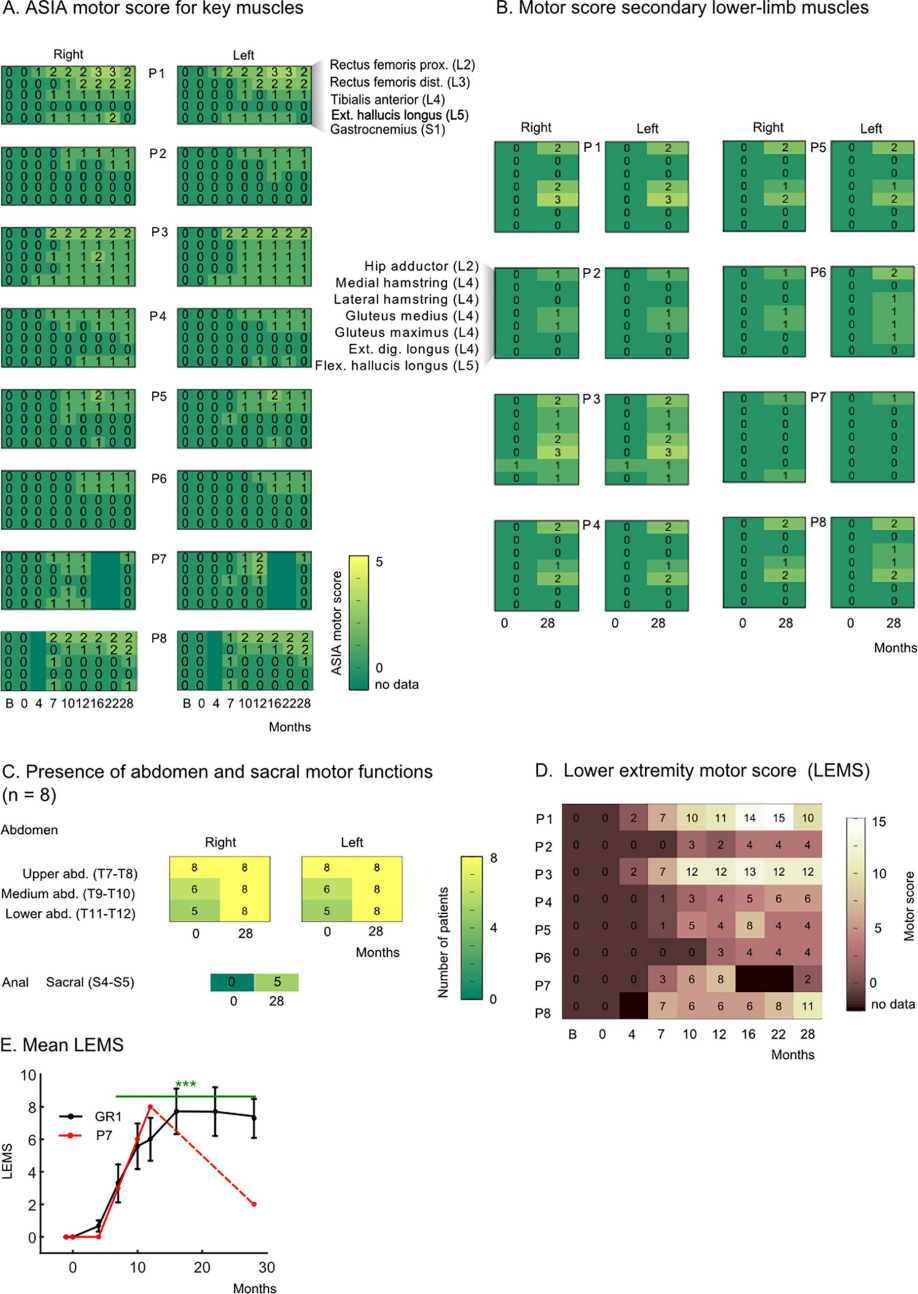
Motor improvement. **Clinical evidence.** (A) Five key lower-limb muscles (proximal and distal rectus femoris, tibialis anterior, extensor hallucis longus and gastrocnemius) were evaluated eight times throughout the training [6] (0 to 28 months) and before starting the training (B: baseline). The motor score describes the amplitude of a contraction from 0 (absence contraction) to 5 (normal contraction). (B) Motor score for seven non-key lower-limb muscles measured at the onset of the training (0) and the end of the end of the training (28 months) for all patients. (C) Number of patients (over eight) with present muscle contraction for three abdominal muscles and the anal sphincter muscle (myotome S4-S5). (D) The LEMS is obtained for each patient by summing the score of all key muscles reported in panel A bilaterally. Missing data periods are in black. (E) Mean± SEM of the motor score for GR1 patients is reported in black and motor score for patient P7 is in red.

Motor functions were tested following the standard AIS evaluation of five key muscles [6] (Fig 4A). To have a complete picture, we assessed seven non-key lower-limb muscles (Fig 4B), three abdominal muscles and the anal sphincter (Fig 4C).

Except for residual contraction in one muscle for patient P3 (motor score of 1 for *extensor digitorium longus*), all patient received a motor score of 0 for the all tested lower-limb muscles (12 per leg) at their admission (baseline measurement). This initial evaluation was confirmed by our medical team at the onset of the training, meaning that, before our training, none of our patients showed any voluntary motor activity in their lower limbs. Throughout the 28 months of training (Fig 4A), we observed continuous motor improvement in the key muscles; with first visible contractions (motor score = 1) and first active movements (without gravity action, motor score = 2) appearing after 7 months of training. After 22 months of training patients, P1 reached a motor score of 3 (active movement against gravity) for the hip flexion (S2 Video).

After 28 months of training, we found active movements (score of 2 and above) in 10 out of 24 tested lower-limb muscles of patient P1, eight muscles for patient P3 and P8, four muscles for patient P4 and P5, and one for patient P6. We also observed motor function recovery in the abdominal muscles, in the three patients who had the highest lesions, namely P4, P5 and P6 (Fig 4C). Finally, in five patients we measured the presence of sphincter control, a muscle whose spinal motor roots originate in the most caudal part of the spinal cord (S4-S5).

The lower extremity motor score (LEMS) was calculated (Fig 4D) by summing the scores for the lower-limb key muscles. The most prominent improvement was observed in the patients with more distal SCI locations (S2, S3 and S4 Videos), namely patients P1, P3 and P8 (lesion range T10-T11, AIS A) (final score respectively 10, 12 and 11). Patients with a more proximal SCI began to show motor improvements after a longer training period: on the 10^th^ month for P2 (T4, AIS B), P4 and P5 (T7-T8, AIS A) and the 12^th^ month for P6 (T4, AIS A). This observed difference in improvement rate among the patients was somewhat expected; the lower-limb muscles are innervated by lumbar and sacral roots. Therefore a patient with a T11 lesion is closer to this level than a patient with a T8 lesion.

As in the case of the sensory recovery, patient P7 displayed an above-average improvement in the motor score during the first year (Fig 4E, red line). However, after protocol discontinuation, he underwent a partial motor regression over the next 16 months, ending up at a level comparable to what he had achieved at the 7th month of training.

Overall, the motor improvement observed in each one of our patients (ranging from 4 to 12 points and, on average, 7.2 points by the end of the protocol) was highly significant when compared to the onset of training (0 for all), and with the spontaneous improvement rates reported in the literature (scores <1 point when the rehabilitation started a year after the injury [12]).

**Fig 5:**
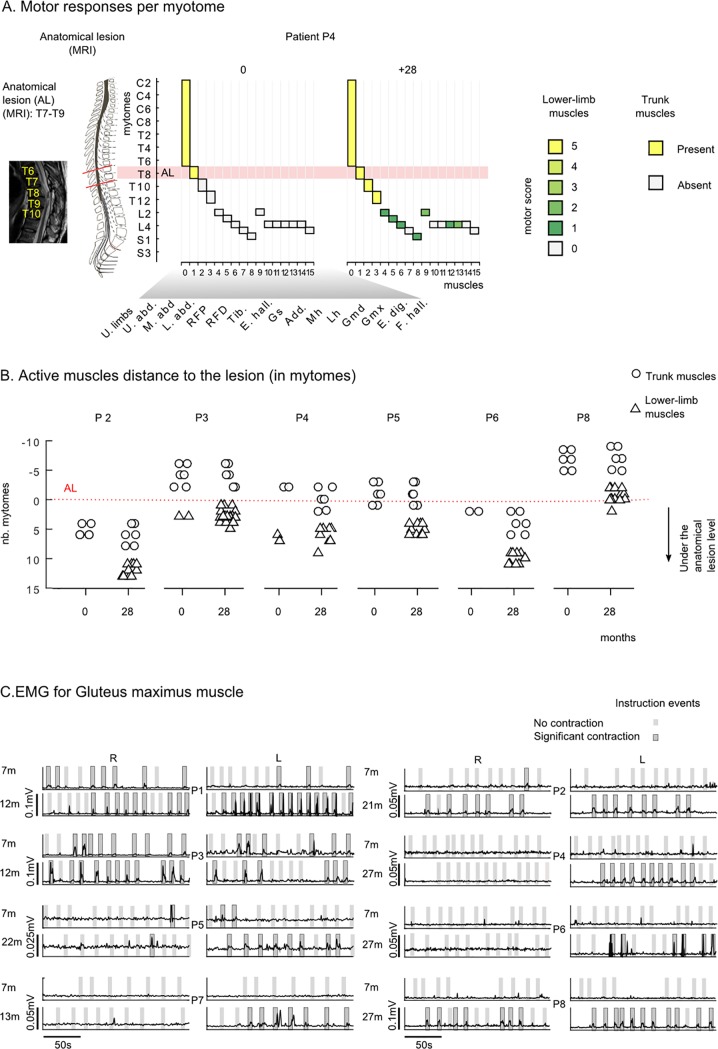
Motor improvement. **Neurological and neurophysiological evidence.** (A) Example of active myotomes compared to the Anatomical Lesion (AL) shown for patient P4. For each muscle, the graphic shows the corresponding myotome level (considering the principal nerve root, S2 Table), and the clinical score at the onset (0) and the end (+28 months) of the WARN training. The MRI of this patient’s spinal cord revealed an AL extending between T7 and T9 segments. Accordingly, at the onset of the training, the patient had preserved motor functions in the upper-limbs and the upper abdomen muscle (spinal nerve roots are located at T7-T8 level), but could not contract the middle and lower abdomen muscles (T9-T12 segment) nor any of the lower limb muscles. After 28 months of the WANR training, this patient had recovered partial motor functions in the middle and lower abdomen as well as in multiple lower limb muscle innervated under the AL, namely, rectus femoris proximal (L2) and distal (L3), hip adductor (L2), gluteus medius/maximus, tibialis anterior (L4), and gastrocnemius (S1). B) For each patient, we considered the muscles where motor functions were clinically observed (ASIA motor score = >1) and calculated the distance to the AL. The distance was calculated as the number of myotomes between the spinal nerve root of the muscle and the lowest segment of the AL. A positive value in the graph, corresponds to a muscle that is rooted below the anatomical lesion; and negative values refer to muscles rooted above the lesion. We report results for the onset (0) and the end of the training (28). Trunk muscles are reported with an open circle, lower limb muscles with an open triangle and upper limb muscles are not considered. (C) EMG envelops for the gluteus maximums muscle for all patients. Patients were instructed to contract their legs for periods of 5 seconds over a 3-minute period. Verbal instructions were given to the patient by the PT; and instruction periods are shown in gray in the graph. A dark gray area highlights the trials where the patient had a significant GMx contraction (> mean + 3xSD of the baseline), and light gray indicates those where the contraction did not reach significance. Muscle responses are shown for all patients (P1 to P8) at an early stage and later in training.

In some cases, the lesion extended over several spinal levels (for example patient P8’s lesion is between T4 and L4). This raised an important question: are all the muscles that recovered activity innervated at the lesion level rather than below? In other words, could our observations be explained by a mechanism of spontaneous recovery at the lesion level of the lower motor neurons (a mechanism known as root recovery [45,46])? To answer this, we calculated, for each patient, the distance between the muscle’s innervation root and the lowest part of the anatomical lesion. For example, patient P4’s MRI scan revealed a lesion at the T7-T9 level. As expected, at the onset of the training all myotomes under the lesion level were silent (Fig 5A). In clear contrast, by the end of the protocol, we observed that recovery extended up to seven spinal levels below the original anatomical lesion (e.g., a level 2 contraction found in the gluteus maximus muscle which is rooted in L4).

We performed the same analysis for patients P2, P3, P5, P6 and P8 (Fig 5B). The anatomical lesion level (AL) for all these patients was obtained through analysis of MRIs (Table 1, S2 Fig). At the onset of the training, we detected very few cases of active myotomes below the AL. A very different result was observed 28 months later (at the protocol’s end), we observed multiple instances of voluntary contractions in muscles that are innervated by spinal nerves that originate below the original AL. For example, for patient P2 we observed contraction in muscles that originated up to 13 spinal segments below the AL; similarly, for patient P3, we identified voluntary muscle contractions five segments below the AL, six for P5, 11 for P6 and two for P8 (Fig 5B). Therefore, we found that the motor improvement in our patients was well below the original SC lesion level and could not be simply explained by a spontaneous root recovery mechanism.

The occurrence of significant motor recovery in all patients was further corroborated through surface EMG recordings of voluntary contractions in multiple lower-limb muscles (Fig 5C). For example, in the case of the gluteus maximus (GMx, hip extensor, L5-S2), we observed a significant increase in contraction force in all patients. The first EMG measurements were obtained 7 months after the training onset. At this point, we did not observe significant contractions in patients P2, P4, P5, P6, and P8. However, after 2 years of training, the same five patients regained the capacity to control GMx voluntarily: P2 and P8 activated GMx bilaterally; P4 and P5 consistently contracted their left GMx. Patient P6 performed less frequent contractions; they were nevertheless aligned with therapist instructions suggesting that the motor activations were voluntary. Patients P1 and P3 experienced the earliest signs of motor recovery; they began producing contractions of their previously paralyzed muscles 7 months after the protocol onset, and contraction force strengthened with continued training. Patient P7 went from 0 to consistent left gluteus contractions between the 7^th^ and 13^th^ month of training.

### Visceral functions improvements

Our patients also exhibited an expressive autonomic function recovery, which was represented by consistent improvements in intestinal, urinary and sexual function, as described in Tables 3–5. Overall, five patients, whose condition changed from AIS A/B to C 28 months after training onset, (P1, P2, P3, P4, P8) regained voluntary anal sphincter motor control (Table 3). Moreover, all seven patients recovered pain and deep pressure sensitivity at the last sacral dermatome, assessed using a pinprick test and the deep anal pressure evaluation, after 2 years of WA-NR training. Consequently, all of them significantly improved their ability to inhibit defecation voluntarily (Table 4). Four patients recovered the ability to experience anal sensation during defecation and, even more importantly for the patients’ quality of life, five regained awareness of the need for defecation.

**Table 3:** ASIA sacral evaluations.

Sacral (ASIA)		0	4	7	10	12	16	22	28
	**Motor**								
	Voluntary anal contraction (S4-S5 myotomes)	0	0	0	1	2	3	4	5
	**Sensitivity**								
Deep anal pressure evaluation (S4-S5 dermatomes)		1	1	1	3	4	4	6	7
Pinprick pain sensitivity (S4-S5 dermatomes)		1	1	1	3	4	4	5	7
**Reflex**									
Pinprick reflex evaluation (S4-S5)		6	6	6	6	6	6	6	7

One motor, two sensory and one reflex tests about the last sacral dermatome/myotome function. Tests measured directly by a physician [6]. Results are shown for eight measurements done throughout the training period (at training onset and after 0, 4, 7, 10, 16, 22, and 28 months) for GR1 patients (n = 7).

**Table 4:** Intestinal function evaluation.

Intestinal	0	4	7	10	12	16	22	28
**Motor**								
Ability to voluntary inhibit defecation	0	0	0	0	1	1	2	5
Fecal incontinence	1	1	1	1	0	0	0	0
**Sensitivity**								
During feces elimination (defecation)	1	2	2	2	2	2	4	4
Awareness of the need for bowel emptying	1	1	1	1	2	2	5	5

Two motor and two sensory tests about the bowel/intestinal functioning for GR1 (n = 7). Tests were collected through a self-questionnaire based on ISCOS data sets [40,41].

**Table 5:** Genitourinary evaluations.

Urinary		0	4	7	10	12	16	22	28
	**Motor**								
	Ability to voluntary avoid urination	0	0	0	0	0	0	1	4
	Involuntary urine leakage	7	5	6	6	6	6	6	3
	**Sensitivity**								
	Awareness of the need for bladder emptying	1	2	1	1	1	1	5	6
	During bladder emptying with catheter	1	1	1	1	1	1	4	5
**Sexual, Genital**		0	4	7	10	12	16	22	28
	**Female**								
	Sensitivity: during sexual intercourse	0	0	0	0	1	1	2	2
	Sensitivity: Menstruation awareness	0	0	0	0	0	0	2	2
	**Male**								
	Sensitivity: During sexual intercourse	1	1	1	1	1	1	1	2
	Motor: psychogenic erection	0	0	0	0	0	0	0	2
	Motor: Reflex	5	5	5	5	5	5	5	5
	Motor: Ejaculation	0	0	0	0	0	0	0	2

Responses to the questionnaire [40,41] related to urinary functions are divided into motor and sensitivity aspects for all patients (n = 7). Questions related to sexual aspects are divided between female (two participants) and male (five participants) patients.

Four patients became capable of voluntarily inhibiting urination (Table 5), while six subjects recovered their ability to perceive the need for bladder emptying and five to perceive the catheter during bladder emptying. Due to these improvements, these patients no longer had to rely on indirect clinical signs, such as sweating, tachycardia and increased spasticity to sense the need to empty their bladders. Such an improvement in urinary functions possibly contributed to the reduction of urinary infections. Indeed, one patient (P6), who experienced repeated cases of lower tract urinary infections before the training onset, exhibited a marked decrease in these events over time: considering the 28 month time range of our protocol, this patient experienced four episodes of lower tract urinary infection during the first year, two during the second year and none during the last 6 months of training. We also observed improvements in sexual and genital function in both female and male patients (Table 5). Both female patients recovered sensitivity during sexual intercourse, as well as awareness and sensitivity to menstruation flow and cramps. While the reflexive erection (controlled by parasympathetic centers S2-S4) was preserved in all male subjects, three of them started experiencing psychogenic erections (physiologically controlled by supraspinal sympathetic centers and thoracolumbar sympathetic spine center at T11-L2), sensitivity during intercourse and ejaculations (conveyed by synergism between supraspinal and spinal centers T11-L2 and S2-S4) functions.

Overall, improvement in bowel, bladder, and sexual function suggested that patients experienced some degree of neurological recovery at the level of sacral segments (S2-S5), which was significantly below the patients’ original SCI.

### Principal factors for sensory and motor recovery

Having documented a marked sensory-motor recovery in our patients, we next investigated which aspects of the WA-NR training protocol (TR) and which external factors (EX) could best account for these partial clinical improvements.

**Fig 6:**
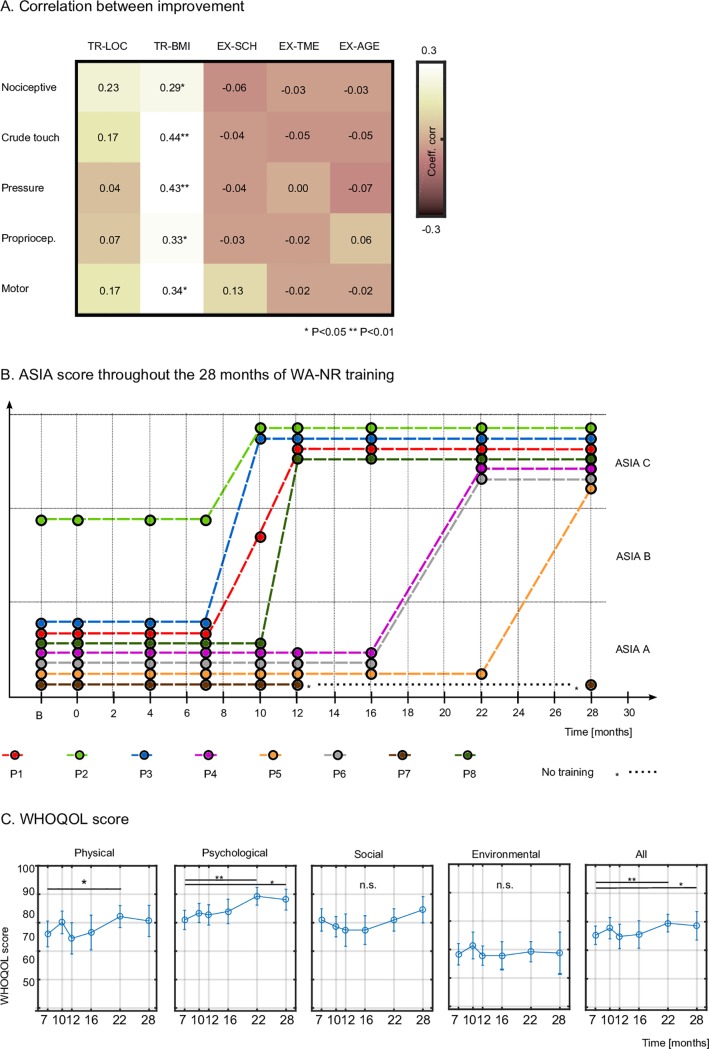
Neurological improvement and correlation with training the protocol. (**A**) The coefficient of correlation between sensory and motor neurological improvements and the number of training hours of active locomotion, BMI-based training, patients SCI height, time since lesion and patients ‘age. (**B**) AIS grade improvement at 28 months. Patient P7 stopped the training after 12 months. A follow-up measurement was done with this patient, 16 months after he stopped the training. (**C**) WHOQOL-BREF [36] score for four subdomains.

Improvement in nociception in our patients was significantly correlated with BMI training hours (Fig 6A, R = 0.29, P = 0.03, Pearson coefficient of correlation), whereas correlation with TR-LOC hours (R = 0.23), did not reach significancy (P = 0.08). Thus, the improvement rate in nociception was most marked during a period with increased BMI-based training. The same was true for the improvement rates for crude touch sensation (R = 0.44, P = 0.002 for TR-BMI, R = 0.17 for TR-LOC, n.s), for pressure improvement (R = 0.43, P = 0.006, versus R = 0.04, n.s.), for proprioception (R = 0.33, P = 0.04 versus R = 0.07, n.s.) as well as for motor improvements (R = 0.34, P = 0.01, for TR-BMI, and 0.17, n.s., for TR-LOC).

Factors like the patients’ age and time since their SCI had no influence on the improvement rate in any of the measured sensory-motor metrics. None of the external factors were correlated with the improvements.

Altogether, all seven patients who remained enrolled in the WA-NR protocol for 28 months changed their initial AIS classification (Fig 6B, see details in S1 Fig). Patients P1, P3, and P8, who had the lowest lesions (T10 –T11), and P2 (AIS B at admission), changed their AIS grade during the first year. Patients P4, P5 and P6, who had higher lesions (T4-T8), changed AIS grade during the second year of training. P7 (T5/T7) was the only patient that stagnated; possibly because he discontinued the training early.

### Quality of life and neuropathic pain

To measure the patients’ quality of life (QoL), the WHOQOL-BREF (World Health Organization Quality of Life Assessment Instrument-Bref) questionnaire was applied six times (first time after 7 months of training) with GR1 patients. Total and partial scores, divided into four domains (physical, psychological, social and environmental) are exhibited in Fig 6C. We observed a significant increase in the physical domain between the 7th (76.0±4.5, mean±SEM) and 22nd months (82.1±3.9) of training (t-test, P<0.05). Similar to the clinical motor score, the WHOQOL physical domain score decreased during the break period between the 10th and 12th months of the protocol (Table 2). In the psychology domain, we found a significant improvement throughout the training (from 80.9±3.38 to 88.13±3.7, considering the seventh and 28^th^ month, t-test, P<0.05). We, however, did not see any significant changes for the social (80.9±3.9 to 84.5±4.6, P>0.1) and environment (68.3±3.9 to 68.7±7.3, P>0.1) domains, the two aspects on which our protocol did not focus.

The details for the physical domain are shown in Table 6. Following training, patients reported better ability in performing their activities of daily living; as well as their capacity for working. This suggests that the training with the WA-NR helped patients reacquire confidence in their ability to work and to achieve higher goals in their lives.

**Table 6:** Mean ± SEM score for all physical domains of the WHOQOL-BREF [36] questionnaire.

WHOQOL physical domains	7	10	12	16	22	28	DIFF (28–7)
**Pain and discomfort**	92.9±4.6	96.4±3.6	89.3±7.4	89.3±7.4	96.4±3.6	92.9±7.1	0.0
**Energy and fatigue**	85.7±5.1	82.1±7.1	64.3±7.4	82.1±7.1	82.1±7.1	89.3±5.1	3.6
**Sleep and rest**	75.0±7.7	71.4±6.5	78.6±6.5	71.4±8.5	78.6±6.5	71.4±10.1	-3.6
**Mobility**	82.1±4.6	75.0±0.0	71.4±3.6	71.4±3.6	78.6±3.6	78.6±6.5	-3.6
**Activities of daily living**	71.4±6.5	78.6±3.6	71.4±8.5	75.0±7.7	82.1±4.6	85.7±5.1	14.3
**Dependence on medication**	64.3±14.3	82.1±10.5	78.6±6.5	78.6±10.1	78.6±11.5	67.9±11.8	3.6
**Work capacity**	71.4±8.5	75.0±5.5	67.9±7.1	67.9±7.1	78.6±3.6	78.6±3.6	7.1

The questionnaire was done respectively after 7,10, 12, 16, 22 and 28 months of training. In the last column, we report the difference between the mean score after 28 months of training and the mean score of the first assessment.

The score for pain and discomfort started and remained positive over the 2 years of the training period; patients did not report ‘that physical pain prevents [them] from doing what [they] need to do.’ For a more detailed measurement of this aspect, the McGill standard evaluation was also applied (Table 7). We considered only the cases of neuropathic (or more precisely, myelopathic) pain like burning/tingling, shocks perceived in areas at the level and under the lesion) and discarded those that were related to external factors (headaches, a postural pain above the lesion level unrelated to the training, etc.). Patient P4, P5, P6, and P7 only presented a few cases of light neuropathic pain (McGill score of 1). Patients P1 and P8 reported a moderate and severe case of pain, respectively, at the onset of the training. The pain reported by P8 was especially uncomfortable, describing it as a sensation of burning in the feet; P1 described moderate pain similar to the sensation of shocks in the leg. The perceived pain attenuated for both patients after following the WANR training, downgrading to 0 for P1 and 1 for P8 (light pain). In one case an increase of neuropathic pain was observed after the onset of the training. Following partial neurological recovery, patient P3 reported a moderate pain in the right thigh.

**Table 7:** McGill score.

	*P1*		*P2*		*P3*		*P4*		*P5*		*P6*		*P7*		*P8*	
**Months of training**	**0**	**28**	**0**	**28**	**0**	**28**	**0**	**28**	**0**	**28**	**0**	**28**	**0**	**12**	**0**	**28**
**Throbbing**	0	**1**	0	0	**1**	**1**	0	**2**	0	0	**1**	0	**1**	**1**	**3**	0
***Shooting***	**2**	0	0	**1**	0	0	0	0	0	0	0	1	1	1	0	0
***Stabbing***	0	0	0	0	0	0	0	0	0	0	0	0	1	0	1	0
***Sharp***	0	0	0	0	0	0	0	0	0	0	0	0	0	0	0	0
***Cramping***	0	0	0	0	0	0	**2**	1	0	0	0	0	0	0	1	0
***Gnawing***	0	0	**1**	0	0	0	0	0	0	0	**1**	0	0	0	0	0
***Hot-burning***	0	0	0	0	0	0	0	**1**	**1**	0	0	1	0	1	**3**	**1**
***Aching***	0	0	0	0	1	**2**	1	0	0	0	0	0	0	0	1	**2**
***Heavy***	0	0	0	0	0	0	0	0	0	0	0	0	0	0	0	0
***Tender***	0	0	0	0	0	**2**	0	1	0	0	0	0	0	0	1	**2**
***Splitting***	0	0	0	0	0	0	0	0	0	0	0	0	0	0	0	0
***Tiring-exhausting***	0	0	0	0	0	0	0	0	0	0	0	0	0	1	1	0
***Sickening***	0	0	0	0	0	0	0	0	0	0	0	0	0	0	**3**	0
***Fearful***	0	0	0	0	0	0	0	0	0	0	0	0	0	0	0	0
***Punishing-cruel***	0	0	0	0	0	0	0	0	0	0	0	0	0	0	0	0
**Sum**	**2**	**1**	**1**	**1**	**2**	**5**	**3**	**5**	**1**	**0**	**2**	**2**	**3**	**4**	**14**	**5**
***Sum neuropathic***	**2**	**1**	**1**	**1**	**1**	**5**	**0**	**1**	**1**	**0**	**2**	**1**	**2**	**2**	**3**	**1**

Detail for the McGill score considering the descriptors for two evaluations, at the onset (0) and at the end of the training (after 12 months for P7 and after 28 months for the other patients). Cases where the descriptors of the pain are below the lesion are considered as neuropathic pain and underlined, those above the lesion (as postural pain, headache, etc.) are not considered to be due to the spinal lesion and therefore not considered for the current analysis.

We found that the patients’ level of fatigue and energy stayed constant during all periods of training and only decreased during the break period (between the 10^th^ and the 12^th^ months); possibly because the WA-NR promoted routine physical training for our patients. Our training did not positively or negatively influence the patients’ sleep. The mobility item measured patients’ autonomy and accessibility to perform their daily life activities (e.g., indoor accessibility.); the item ‘dependence on medication’ measured patients general use of medication (considering both chronic and acute pain). As expected, our training did not influence these two items.

Concerning the psychological domain, we documented an improvement in five out of the six sub-items (Table 8). Patients’ reported enjoying their lives more (an increase of 7.1 for positive feelings), corroborated with a decrease in the occurrence of negative feelings, such as blue mood, despair, anxiety, and depression (improvement of 10.7 points). Patients reported being more satisfied with themselves (+7.1 point in self-esteem) reaching a very high score by the end of the training (96.4%). We believe that the patients’ improvement in the thinking/concentration/memory item reflected the fact that training with the BMI specifically required subjects to focus on their rehabilitation tasks. Several studies have shown that BMI training is beneficial for improving focus and concentration [47]. Also, we observed that body-image plateaued at a high score throughout the training (~90%). Finally, one of the most interesting results came from asking whether patients ‘[..]feel [their] life to be meaningful’ (spiritual/personal beliefs). We observed a continuous improvement over the training period, suggesting that the participation in the WA-NR protocol had a positive impact on the patients’ self-esteem.

**Table 8:** Mean ± SEM score for all psychological domains of the WHOQOL-BREF [36] questionnaire.

WHOQOL psychology domains	7	10	12	16	22	28	DIFF (28–7)
**Positive feelings**	71.4±3.6	78.6±3.6	75.0±5.5	75.0±5.5	82.1±4.6	78.6±6.5	7.1
**Self-esteem**	89.3±5.1	85.7±5.1	89.3±5.1	89.3±5.1	96.4±3.6	96.4±3.6	7.1
**Thinking, memory and concentration**	82.1±7.1	82.1±4.6	82.1±4.6	78.6±6.5	89.3±5.1	89.3±5.1	7.1
**Bodily image and appearance**	89.3±5.1	89.3±5.1	85.7±5.1	92.9±4.6	92.9±4.6	89.3±5.1	0.0
Negative feelings*	78.6±3.6	85.7±5.1	85.7±5.1	89.3±5.1	92.9±4.6	89.3±5.1	10.7
**Spirituality/personal beliefs**	75.0±5.5	78.6±3.6	78.6±6.5	78.6±8.5	82.1±4.6	85.7±5.1	10.7

The questionnaire was done respectively after 7,10, 12, 16, 22 and 28 months of training. The difference of mean score after 28 months as compared to the mean score of the first assessment.

* High scores mean fewer negative feelings.

## Discussion

The present study reports a systematic and unprecedented partial neurological recovery in patients diagnosed with chronic complete (AIS A) and motor complete (AIS B) paraplegia, following long-term non-invasive neurorehabilitation [33]. Based on a study that gathered clinical data from a group of eight SCI patients over a 28-month period, we found that the longer the patients trained under the protocol that combined a BMI, visuo-tactile feedback and active locomotion, the larger was the sensory-motor and visceral recovery observed below the SCI level. This was true for both somatosensory (tactile, nociceptive, proprioceptive, pressure and vibration), motor (voluntary contraction observed for multiple myotomes below the original lesion), and autonomic functions (sexual, intestinal and urinary).

At the core of the WA-NR protocol, concurrent BMI-based control of virtual and mechanical actuators combined brain activation with continuous visuo-tactile feedback and physical training, assisted by a body weight support system and robotic gait therapy devices. Thus, the principal difference between the WA-NR protocol and other existing neurorehabilitation paradigms is that it focuses neither on the physical, nor on the BMI approach *per se*, but instead it creates the conditions to simultaneously engage both cortical and peripheral signals that converge towards the level of the SCI. As such, the intended goal is to reinforce the potential physiological role played by spinal tracts that have survived the original injury.

Depending on the cause, a traumatic SCI can lead to a variety of lesions, generated by contusion, compression or penetration, which is followed by massive necrosis of affected neural circuits during the first 18 hours post-injury [48]. During the subsequent weeks, the neuroimmunological system deflagrates a cascade of mechanisms that may expand the lesion above and below its original epicenter. Cavity or cyst formation and demyelination may also occur, damaging both ascending and descending pathways [48].

Previous studies have shown that 84% of clinically complete paraplegics (AIS A) exhibit some neurophysiological activity below the level of the injury [7,49] and are therefore referred to as “discomplete” SCI [8]; post-mortem analysis by Kakulas et al. [9] confirmed the presence of fibers in patients diagnosed as having a clinically complete injury. Confirming this assessment, MRI scans of our patients showed the residual spinal cord continuity in three out of the six patients (two AIS A, one AIS B).

Based on these new observations, we suggest that even a small portion of surviving spinal cord axons can contribute to a meaningful clinical and functional recovery, provided that they can be properly re-engaged by a long-term rehabilitation protocol like ours. In support of this point of view, animal models of SCI have shown that sparing of about 10–15% of the spinal cord is sufficient to support a partial recovery of locomotion [50]. In human subjects, the exact amount of spared SC white matter needed to observe similar levels of functional neurological recovery remains unknown. However, in a study in which a cordotomy was performed in 44 SCI patients [51] to alleviate secondary cancer-induced pain, Nathan et al. showed that even a complete bilateral section of the anterior portion of the spinal cord, containing motor tracts, did not significantly affect patients’ motor functions. They suggested that the motor tracts in the posterior half of the spinal cord could compensate for the functions originally mediated by the anterior portion.

Our studies confirm the role played by the employment of BMI-based training to induce both partial sensory and motor recoveries. We found that periods of increased hours of BMI-based training yielded the most pronounced clinical improvements. Importantly, we also observed that the improvement rate was dependent neither on the period since the patients’ original SCI, nor the patients’ age.

But what mechanism could account for this recovery? We hypothesize that by triggering an extensive process of cortical and spinal cord functional plasticity, our BMI paradigm created the conditions for our patients to recover sensory-motor and visceral functions. These results are in accordance with recent observations in a rat model [27,31]. Moreover, our results indicate that both neurological and local muscular factors contribute to the final motor outcome following the WA-NR protocol. Indications that recovery is happening due to neurological factors include a proximal-to-distal order of recovery. The L2 -innervated muscles had voluntary contractions in all eight patients, while more distal levels like L5 were seen in two patients and S1 in one patient only. If there were only neurological factors involved in the final outcome, it should be expected that all muscles innervated by a nerve root would respond equally, which is not the case. The analysis of L4-innervated muscles allows us to compare the tibialis anterior (key-muscle) with five other muscles (gluteus maximus, gluteus medius, medial hamstring, lateral hamstring, and extensor digitus longus) which differ in size and location. The glutei have shown better scores among the majority of patients, while hamstrings and extensor digitus longus had poor responses. Tibialis anterior, which is a key-muscle for L4 evaluation had lower scores than the glutei, showing that non-key muscle testing is indeed important to rule out inhomogeneities of recovery due to local muscular factors.

Previously, BMI-evoked cortical plasticity has been reported in the rehabilitation of stroke patients (36). These studies have shown a considerable clinical effect of BMI training even in patients with severe neurological impairment. Similar to the classical physical therapy protocols for incomplete SCI [52], repeated active motor tasks, which promote activity-dependent rehabilitation, have been recognized for inducing partial motor recovery in stroke patients [53,54]. Constraint-induced movement therapy, for example, has been successfully used for stroke rehabilitation, even at the chronic phase [55]. However, this approach relies on the utilization of the patient’s residual motor functions, a condition that is absent in 30–50% of stroke victims [56]. Therefore, BMI-based therapy was adopted for these most severe stroke cases, in which no residual motor function was present. This method aimed at rehearsing a lost motor function, through real-time decoding of the patient’s motor imagery and online feedback (visual or proprioceptive [57,58]). In this approach, the BMI serves as a cortical operant conditioning (as shown in pioneer work of Fetz and collaborators [59]). We believe that a similar neurophysiological mechanism was activated in our SCI patients when they were subjected to the WA-NR protocol. At the onset of the training, our patients, who were diagnosed with complete paraplegia, could not follow classical training with the active motor task. Instead, our BMI-based neurorehabilitation, which integrated decoding of cortical motor imagery with visuo-tactile feedback, provided the driving force for triggering the kind of cortical plastic process that mediated their recovery.

Concomitant to a partial somatosensory and motor recovery, we also demonstrated that training with the WA-NR protocol induced a significant change in our patients’ perception of their own bodies, as evident from the partial recovery of tactile, proprioceptive and vibratory sensitivity. Thus, our protocol affected the patients’ body schema, which involves the multisensory integration of visual, kinesthetic and proprioceptive information to provide humans with a sense of being and existing in space [60,61]. Previous studies have shown that the body schema is plastic and, hence, can become distorted after SCI [62,63] or a limb amputation [64]. At training onset, patients did not detect the presence of vibration stimulation in their lower limbs. When they first began perceiving tactile sensations from the parts of their body that had remained numb since their original SCI (i.e., for many years), their perception of the tactile stimuli location was distorted. As a rule, after the patients began to regain sensations in their legs, they tended to perceive vibration in more distal parts of the leg, compared to the actual stimulation site.

One explanation of this effect is the sensory stimulation activated not only the cortical areas representing the stimulated body part, but it also invaded adjacent representations of the more distal parts that had remained silent for a long time. Such “filling-in” of sensory deprived areas of the primary somatosensory cortex has been previously reported in animal models [65] and amputee patients [66,67]. But, the observation of a constant number of misplacement errors toward the opposite leg, an area not adjacent in the cortical somatotopic representation, suggests additional mechanisms to the classic ‘invading’ representation explanation. A possible explanation is that the peripheral information entered the somatotopic representation of the stimulated body part but was interpreted incorrectly because of the body schema distortion caused by many years of sensory deprivation.

Following the sensory recovery in the lower-limbs, we observed that at first the patients could detect the presence of stimulation but their spatial representation was distorted, and then, the sensory map became more organized. We believe that the extensive training using congruent visual, tactile and proprioceptive signals, was essential for patients to recover a more structured body representation. Indeed, we have demonstrated in a previous study that the congruent use of the virtual reality and tactile feedback setup used in the WA-NR induced a significant change of body schema representation in SCI patients [35], in coherence with numerous studies showing this effect with healthy subjects [68,69]. Overall, we propose that the temporal evolution of our patients’ perception of their bodies emerged from a complex process of activity-dependent plasticity, occurring in the body representations that exist at the cortical level, which altogether define the patient’s body schema [60]. Likely, this cortical plasticity was paralleled by a similar process taking place at subcortical levels, both of which were influenced by peripheral contributions to this body representation. In addition to lower limb sensory-motor dysfunction, the most devastating effects of SCI are genitourinary, gastrointestinal and sexual dysfunction [70]. Surveys have shown that paraplegic patients ranked as their first priority the recovery of sexual functions (26%), followed by bladder/bowel functions (18%), before movement and sensation recovery (16% and 7.5%) [71]. In our study, five out of the seven patients became aware of the need for bowel emptying and recovered the ability to inhibit voluntary defecation 22–28 months after the training onset. In addition, four patients regained the ability to inhibit urination and six recovered awareness of the need for bladder emptying. These changes had a very positive impact on the patients’ health, as they reduced the risk of patients acquiring a urinary infection and exposure to autonomic dysreflexia that could lead to uncontrolled hypertension. Significant gains in the patients’ ability to inhibit urination also gave them more flexibility in their daily activities and improved the social interactions.

Another fundamental observation of this long-term study was that four patients (two males and two females) partially recovered their sexual functions. This observation suggests that the partial neurological recovery obtained was not constrained to the lower-limb somatosensory and motor functions, which were the primary focus of our BMI and the physical training, but, instead, resulted from a generalized neurological recovery, which also manifested itself as an improvement in major visceral functions.

Although the rate of clinical improvement of the patients was higher during the first year of training compared to the second, the patients’ recovery continued to improve for the 28 months of training. Yet, it is not entirely clear at this point to what extent the effects of WA-NR are preserved after a patient stops this training. The results for one patient showed that, after displaying promising clinical improvements, above the group average, during the first 12 months of training, this patient maintained the majority of his sensory gains for the remaining 18 months, even after he discontinued training. This was true for the rate of improvement for tactile, nociceptive, proprioception and pressure sensitivity modalities, but not for vibration. Moreover, upon discontinuation, the same patient exhibited a clear regression of motor functions gained during the first year of training but did not return to his starting point (which was equal to zero at the protocol onset). These observations indicate the importance of continued engagement with the WA-NR protocol. Longer follow-up periods with more patients will be needed to evaluate the long-term effects of discontinued training on the partial gains in sensory, motor, and visceral functions obtained with the WA-NR protocol.

During our research, the patients had integral psychological support with the purpose of guiding expectations adjustments, regarding the neurological improvements that the patients could potentially exhibit. Our major concern was the impact of the walking training on patients, as subjects were previously prepared to independently perform activities of daily living (ADL) in a wheelchair and were already adapted to this condition. An important question that was raised in this study was how the training would impact the patients’ own subjectivity and change (if it does) their quality of life (QoL) perception. QoL is not merely the absence of disease, but the state of complete physical, psychological and social wellbeing. In recent years, QoL improvement for SCI has become a rehabilitation goal [72,73], and its assessment is considered beneficial for evaluating the outcome of a multidisciplinary rehabilitation team approach. The patient’s self-perception of the QoL is considered an efficacy measurement of treatment and can also be used to evaluate the cost-effectiveness of interventions, and research and rehabilitation programs [74]. In our study, we observed increases in total, physical and psychological subdomains of QoL following our protocol. The physical domain exhibited fast improvement at the onset of the training, and a decrease during the break period, stressing the importance of maintaining continuous rehabilitation activities in these patients.

Unlike changes observed in the physical domain, the psychological domain exhibited a gradual improvement and a long-term effect on the course of training with the WA-NR protocol. While partial neurological recovery can contribute to an improvement in QoL in spinal cord injury patients [75], we suggest that another important factor can explain the positive effect observed during the execution of the WA-NR protocol: patients were fully engaged in their training as they were encouraged to imagine themselves performing movements while they received rich visual, vibrotactile and proprioceptive feedback; in other words, patients were required to be active protagonists of their physical therapy and neurorehabilitation process.

As part of our follow-up assessing the patients’ quality of life, we also controlled for instances of neuropathic pain. Below-lesion neuropathic pain (NP) is present in 34% of SCI patients [76] and can significantly reduce subjects’ quality of life and compromise the neurorehabilitation process [77]. In our case, as confirmed by WHOQOL results, we did not observe cases of NP that prevented patients from performing their training normally. However, in one case a severe NP reported at the onset of the protocol, and described as a sensation of burning in the feet, was later alleviated. We propose here a hypothesis for this mechanism of reduction of NP. Motor cortex (M1) is thought to play an important role in modulation of pain [78–80]; indeed stimulation of M1 is a well-studied treatment for chronic NP especially after deafferentation (post-stroke pain, brachial plexus avulsion, phantom limb pain and also post spinal cord injury NP). By promoting higher activation in the sensory-motor cortical areas, the BMI may have played an important role in the reduction of NP. Indeed, in a case study with one chronic SCI patient, 4 months of training with a non-invasive BMI was shown to promote a reduction in neuropathic pain [81]). Further studies with a group of patients with higher levels of neuropathic pain are necessary to investigate this hypothesis in a more comprehensive way.

Overall, our patients’ training with the WA-NR promoted a sense of empowerment [82] and acted by strengthening the sense of competence, self-worth, and self-esteem of our patients. It allowed the individual to overcome the situation of helplessness and develop control over their own lives. The concept of patients’ empowerment has been gathering interest in various health care domains [83–88] since it promotes the concept of self-determination of patients as agents of their health and healthcare [89]. We suggest that the empowerment component may have influenced the perception of our patients’ capacity to contribute to their own recovery and, consequently, help improve their QoL. This process generates a sense of autonomy for patients, as they realized that they were actively involved in seeking some degree of clinical improvement. This improvement resonated in patients’ daily habits, as, for example, their dressing habit; for the first time since the lesion, two patients started using shorter cloth (skirts/shorts) revealing parts of their body they had been hiding.

Overall, our long-term clinical results open new therapeutic perspectives for the rehabilitation of the most severe cases of SCI (AIS A and B), even at the chronic phase, while using a purely non-invasive BMI-based approach. Therefore, our findings suggest that existing or future technologies, created for incomplete AIS C patients, may also be used for patients originally classified as AIS A/B. For example, a large number of existing orthoses and exoskeletons, which require lower limb EMGs for their actuation (see [90] for a review), could now be potentially considered for use with AIS A patients, following a period of BMI-based training, using a protocol like the WA-NR. Our findings also indicate that, given a small fraction of spared spinal cord white matter, a much larger than expected population of AIS A/B patients might benefit from neurorehabilitation protocols that actively engage the patients’ mental and physical activity, while providing them with rich visual and tactile feedback.

## Supporting information

S1 Fig ASIA score sheet for all patients at onset and at the end of the training. (PDF)

S2 Fig MRI of patients’ spinal cord. (**A**) MRI cuts of sagittal and (**B**) axial planes (T2 sequence) at SCI level for patients P2, P3, P4. Myelomalacia (hyperintense signal, 7mm length) is visible for patient P2 at the level of thoracic vertebra T2, and continuity of neural fibers are visible at the lesion level. For patient P3, we observed the spinal cord injury extending between thoracic vertebras T10 and L1 with the presence of remaining fibers (dark gray). T12 axial plane for the same patient reveals injury arachnoiditis (inflammation of spinal meninges) and fiber continuity (better visualization). For patient P4, close to the vertebral body fracture at T8-T9 level, we also observe neural fiber continuity; axial plane reveals dural sac septations at injury level and fiber continuity. (**C**) Example of 3D segmentation and projection on sagittal and coronal plans for patient P4, confirms spinal cord continuity at lesion level.
(PDF)

S1 Video 3D MRI reconstruction. Three-dimensional reconstruction based on FIESTA sequence images for patient P4. The rendering was done with OsiriX Lite software. Published with permission of Associação Alberto Santos Dumont para Apoio à Pesquisa (AASDAP), Sao Paulo, Brazil.
(MP4)

S2 Video Motor examination in a suspended position for patient P1. The motor exam was done 9 and 22 months after the onset of the training. The patient is instructed to flex the right hip. Published with permission of Associação Alberto Santos Dumont para Apoio à Pesquisa (AASDAP), Sao Paulo, Brazil.
(MP4)

S3 Video ASIA motor examination of patient P3. The motor exam is done 32 months after the onset of the training. The patient is asked to align the lower limbs, performing hip adduction and knee extension, for the left side and later for the right side. Published with permission of Associação Alberto Santos Dumont para Apoio à Pesquisa (AASDAP), Sao Paulo, Brazil.
(MP4)

S4 Video Motor examination in a suspended position for patient P8. The motor exam was done 29 months after the training onset. The patient was instructed to move both legs alternatively backward (right and later left side) and forward (right and later left side); with 75–80% of body weight support during forward movement and 65–70% during backward movement. Is possible to see EMGs electrodes placed at the lower limbs, for neurophysiology analysis, and the hands of a therapist are stabilizing the pelvis, avoiding body rotations. Published with permission of Associação Alberto Santos Dumont para Apoio à Pesquisa (AASDAP), Sao Paulo, Brazil.
(MP4)

S1 Table WHOQOL-BREF set of questions per domain. (DOCX)

S2 Table For each muscle, the corresponding nerve, nerve root range. The reported principal nerve is the one reported in ASIA assessment (except extensor digitorum longus which is not part of the ASIA assessment).
(DOCX)

S3 Table Patient’s proprioception score per movement after 28 months of training. (DOCX)
