# Supplementary material for: Training with brain-machine interfaces, visuo-tactile feedback and assisted locomotion improves sensorimotor, visceral, and psychological signs in chronic paraplegic patients
Source: PLoS One. 2018 Nov 29;13(11):e0206464. doi: 10.1371/journal.pone.0206464 (PMC6264837; doi:10.1371/journal.pone.0206464)
Supplement: S1 Fig — (PDF) [file pone.0206464.s001.pdf]

P1

Onset

Onset	Pin prick(PP) R	Pin prick(PP) L	Light touch (LT) R	Light touch (LT) L
**C2**	2	2	2	2
**C3**	2	2	2	2
**C4**	2	2	2	2
**C5**	2	2	2	2
**C6**	2	2	2	2
**C7**	2	2	2	2
**C8**	2	2	2	2
**T1**	2	2	2	2
**T2**	2	2	2	2
**T3**	2	2	2	2
**T4**	2	2	2	2
**T5**	2	2	2	2
**T6**	2	2	2	2
**T7**	2	2	2	2
**T8**	2	2	2	2
**T9**	2	2	2	2
**T10**	2	2	2	2
**T11**	2	2	2	1
**T12**	1	0	1	0
**L1**	0	0	0	0
**L2**	0	0	0	0
**L3**	0	0	0	0
**L4**	0	0	0	0
**L5**	0	0	0	0
**S1**	0	0	0	0
**S2**	0	0	0	0
**S3**	0	0	0	0
**S4-S5**	0	0	0	0
Total	**37**	**36**	**37**	**35**

Motor		Right	Left
Elbow Flexor	C5	5	5
Wrist extensor	C6	5	5
Elbow extensor	C7	5	5
Finger flexor	C8	5	5
Finger abductors	T1	5	5
Hip flexors	L2	0	0
Knee exntensors	L3	0	0
Ankle dorsiflexors	L4	0	0
Long toes extensors	L5	0	0
Ankle plantar flexors	S1	0	0
	Total	**25**	**25**

(VAC) Voluntary anal contraction (yes/no): No

Deep Anal Pressure: No

Motor score

UER	**25**	Max (25)
UEL	**25**	(25)
UEMS TOTAL	**50**	(50)
LER	**0**	Max (25)
LEL	**0**	(25)
TOTAL LEMS	**0**	(50)

Sensory Subscores

RLT	**37**	MAX (56)
LLT	**35**	(56)
LT TOTAL	**72**	(112)
RPP	**37**	MAX (56)
LPP	**36**	(56)
PP TOTAL	**73**	(112)

NEUROLOGICAL LEVELS

	R	L
Sensory	T11	T10
Motor	T11	T10

Neurological level of injury (NLI): **T10**

Complete or incomplete: **C**

Impairment scale: A

Zone of partial preservation

	R	L
Sensory	T11	T10
Motor	T11	T10

End

End	Pin prick(PP) R	Pin prick(PP) L	Light touch (LT) R	Light touch (LT) L
**C2**	2	2	2	2
**C3**	2	2	2	2
**C4**	2	2	2	2
**C5**	2	2	2	2
**C6**	2	2	2	2
**C7**	2	2	2	2
**C8**	2	2	2	2
**T1**	2	2	2	2
**T2**	2	2	2	2
**T3**	2	2	2	2
**T4**	2	2	2	2
**T5**	2	2	2	2
**T6**	2	2	2	2
**T7**	2	2	2	2
**T8**	2	2	2	2
**T9**	2	2	2	2
**T10**	2	2	2	2
**T11**	2	2	2	2
**T12**	2	2	2	2
**L1**	2	1	2	1
**L2**	1	1	1	1
**L3**	0	0	0	0
**L4**	0	0	0	0
**L5**	0	0	0	0
**S1**	0	0	0	0
**S2**	0	0	0	0
**S3**	0	0	0	0
**S4-S5**	1	1	0	0
	**42**	**41**	**41**	**40**

Motor		Right	Left
Elbow Flexor	C5	5	5
Wrist extensor	C6	5	5
Elbow extensor	C7	5	5
Finger flexor	C8	5	5
Finger abductors	T1	5	5
Hip flexors	L2	2	2
Knee exntensors	L3	2	2
Ankle dorsiflexors	L4	1	1
Long toes extensors	L5	0	0
Ankle plantar flexors	S1	0	0
	Total	**30**	**30**

(VAC) Voluntary anal contraction (yes/no): Yes

Deep Anal Pressure: Yes

Motor score

UER	**25**	Max (25)
UEL	**25**	(25)
UEMS TOTAL	**50**	(50)
LER	**5**	Max (25)
LEL	**5**	(25)
TOTAL LEMS	**10**	(50)

Sensory Subscores

RLT	**41**	MAX (56)
LLT	**40**	(56)
LT TOTAL	**81**	(112)
RPP	**42**	MAX (56)
LPP	**41**	(56)
PP TOTAL	**83**	(112)

NEUROLOGICAL LEVELS

	R	L
Sensory	L1	T12
Motor	T12	T12

Neurological level of injury (NLI): **T12**

Complete or incomplete: **I**

Impairment scale: C

Zone of partial preservation

	R	L
Sensory		
Motor		

P2

Onset

Onset	Pin prick(PP) R	Pin prick(PP) L	Light touch (LT) R	Light touch (LT) L
**C2**	2	2	2	2
**C3**	2	2	2	2
**C4**	2	2	2	2
**C5**	2	2	2	2
**C6**	2	2	2	2
**C7**	2	2	2	2
**C8**	2	2	2	2
**T1**	2	2	2	2
**T2**	2	2	2	2
**T3**	2	2	2	2
**T4**	2	2	2	2
**T5**	1	1	1	1
**T6**	1	1	1	1
**T7**	1	1	1	1
**T8**	1	1	1	1
**T9**	1	1	1	1
**T10**	1	1	1	1
**T11**	1	1	1	1
**T12**	1	1	1	1
**L1**	1	1	1	1
**L2**	1	1	1	1
**L3**	1	1	1	1
**L4**	1	1	1	1
**L5**	1	1	1	1
**S1**	1	1	1	1
**S2**	1	1	1	1
**S3**	1	1	1	1
**S4-S5**	1	1	1	1
Total	**39**	**39**	**39**	**39**

Motor		Right	Left
Elbow Flexor	C5	5	5
Wrist extensor	C6	5	5
Elbow extensor	C7	5	5
Finger flexor	C8	5	5
Finger abductors	T1	5	5
Hip flexors	L2	0	0
Knee exntensors	L3	0	0
Ankle dorsiflexors	L4	0	0
Long toes extensors	L5	0	0
Ankle plantar flexors	S1	0	0
	Total	**25**	**25**

(VAC) Voluntary anal contraction (yes/no): No

Deep Anal Pressure: Yes

Motor score

UER	**25**	Max (25)
UEL	**25**	(25)
UEMS TOTAL	**50**	(50)
LER	**0**	Max (25)
LEL	**0**	(25)
TOTAL LEMS	**0**	(50)

Sensory Subscores

RLT	**39**	MAX (56)
LLT	**39**	(56)
LT TOTAL	**78**	(112)
RPP	**39**	MAX (56)
LPP	**39**	(56)
PP TOTAL	**78**	(112)

NEUROLOGICAL LEVELS

	R	L
Sensory	T4	T4
Motor	T4	T4

Neurological level of injury (NLI): **T4**

Complete or incomplete: **I**

Impairment scale: B

Zone of partial preservation

	R	L
Sensory		
Motor		

End

End	Pin prick(PP) R	Pin prick(PP) L	Light touch (LT) R	Light touch (LT) L
**C2**	2	2	2	2
**C3**	2	2	2	2
**C4**	2	2	2	2
**C5**	2	2	2	2
**C6**	2	2	2	2
**C7**	2	2	2	2
**C8**	2	2	2	2
**T1**	2	2	2	2
**T2**	2	2	2	2
**T3**	2	2	2	2
**T4**	2	2	2	2
**T5**	2	2	2	2
**T6**	1	1	1	1
**T7**	1	1	1	1
**T8**	1	1	1	1
**T9**	1	1	1	1
**T10**	1	1	1	1
**T11**	1	1	1	1
**T12**	1	1	1	1
**L1**	1	1	1	1
**L2**	1	1	1	1
**L3**	1	1	1	1
**L4**	1	1	1	1
**L5**	1	1	1	1
**S1**	1	1	1	1
**S2**	1	1	1	1
**S3**	1	1	1	1
**S4-S5**	1	1	1	1
Total	**40**	**40**	**40**	**40**

Motor		Right	Left
Elbow Flexor	C5	5	5
Wrist extensor	C6	5	5
Elbow extensor	C7	5	5
Finger flexor	C8	5	5
Finger abductors	T1	5	5
Hip flexors	L2	1	1
Knee exntensors	L3	1	1
Ankle dorsiflexors	L4	0	0
Long toes extensors	L5	0	0
Ankle plantar flexors	S1	0	0
	Total	**27**	**27**

(VAC) Voluntary anal contraction (yes/no): Yes

Deep Anal Pressure: Yes

Motor score

UER	**25**	Max (25)
UEL	**25**	(25)
UEMS TOTAL	**50**	(50)
LER	**2**	Max (25)
LEL	**2**	(25)
TOTAL LEMS	**4**	(50)

Sensory Subscores

RLT	**40**	MAX (56)
LLT	**40**	(56)
LT TOTAL	**80**	(112)
RPP	**40**	MAX (56)
LPP	**40**	(56)
PP TOTAL	**80**	(112)

NEUROLOGICAL LEVELS

	R	L
Sensory	T5	T5
Motor	T5	T5

Neurological level of injury (NLI): **T5**

Complete or incomplete: **I**

Impairment scale: C

Zone of partial preservation

	R	L
Sensory		
Motor		

P3

Onset

Onset	Pin prick(PP) R	Pin prick(PP) L	Light touch (LT) R	Light touch (LT) L
**C2**	2	2	2	2
**C3**	2	2	2	2
**C4**	2	2	2	2
**C5**	2	2	2	2
**C6**	2	2	2	2
**C7**	2	2	2	2
**C8**	2	2	2	2
**T1**	2	2	2	2
**T2**	2	2	2	2
**T3**	2	2	2	2
**T4**	2	2	2	2
**T5**	2	2	2	2
**T6**	2	2	2	2
**T7**	2	2	2	2
**T8**	2	2	2	2
**T9**	2	2	2	2
**T10**	2	2	2	2
**T11**	1	2	1	2
**T12**	0	1	0	1
**L1**	0	0	0	0
**L2**	0	0	0	0
**L3**	0	0	0	0
**L4**	0	0	0	0
**L5**	0	0	0	0
**S1**	0	0	0	0
**S2**	0	0	0	0
**S3**	0	0	0	0
**S4-S5**	0	0	0	0
	**35**	**37**	**35**	**37**

Motor		Right	Left
Elbow Flexor	C5	5	5
Wrist extensor	C6	5	5
Elbow extensor	C7	5	5
Finger flexor	C8	5	5
Finger abductors	T1	5	5
Hip flexors	L2	0	0
Knee exntensors	L3	0	0
Ankle dorsiflexors	L4	0	0
Long toes extensors	L5	0	0
Ankle plantar flexors	S1	0	0
	Total	**25**	**25**

(VAC) Voluntary anal contraction (yes/no): No

Deep Anal Pressure: No

Motor score

UER	**25**	Max (25)
UEL	**25**	(25)
UEMS TOTAL	**50**	(50)
LER	**0**	Max (25)
LEL	**0**	(25)
TOTAL LEMS	**0**	(50)

Sensory Subscores

RLT	**35**	MAX (56)
LLT	**37**	(56)
LT TOTAL	**72**	(112)
RPP	**35**	MAX (56)
LPP	**37**	(56)
PP TOTAL	**72**	(112)

NEUROLOGICAL LEVELS

	R	L
Sensory	T10	T11
Motor	T10	T11

Neurological level of injury (NLI): **T10**

Complete or incomplete: **C**

Impairment scale: A

Zone of partial preservation

	R	L
Sensory	T10	T11
Motor	T10	T11

End

End	Pin prick(PP) R	Pin prick(PP) L	Light touch (LT) R	Light touch (LT) L
**C2**	2	2	2	2
**C3**	2	2	2	2
**C4**	2	2	2	2
**C5**	2	2	2	2
**C6**	2	2	2	2
**C7**	2	2	2	2
**C8**	2	2	2	2
**T1**	2	2	2	2
**T2**	2	2	2	2
**T3**	2	2	2	2
**T4**	2	2	2	2
**T5**	2	2	2	2
**T6**	2	2	2	2
**T7**	2	2	2	2
**T8**	2	2	2	2
**T9**	2	2	2	2
**T10**	2	2	2	2
**T11**	2	2	2	2
**T12**	1	2	1	2
**L1**	1	1	1	2
**L2**	0	0	0	0
**L3**	0	0	0	0
**L4**	0	0	0	0
**L5**	0	0	0	0
**S1**	0	0	0	0
**S2**	0	0	0	0
**S3**	0	0	0	0
**S4-S5**	1	1	1	1
	**39**	**40**	**39**	**41**

Motor		Right	Left
Elbow Flexor	C5	5	5
Wrist extensor	C6	5	5
Elbow extensor	C7	5	5
Finger flexor	C8	5	5
Finger abductors	T1	5	5
Hip flexors	L2	2	2
Knee exntensors	L3	1	1
Ankle dorsiflexors	L4	1	1
Long toes extensors	L5	1	1
Ankle plantar flexors	S1	1	1
	Total	**31**	**31**

(VAC) Voluntary anal contraction (yes/no): Yes

Deep Anal Pressure: Yes

Motor score

UER	**25**	Max (25)
UEL	**25**	(25)
UEMS TOTAL	**50**	(50)
LER	**6**	Max (25)
LEL	**6**	(25)
TOTAL LEMS	**12**	(50)

Sensory Subscores

RLT	**39**	MAX (56)
LLT	**41**	(56)
LT TOTAL	**80**	(112)
RPP	**39**	MAX (56)
LPP	**40**	(56)
PP TOTAL	**79**	(112)

NEUROLOGICAL LEVELS

	R	L
Sensory	T11	T12
Motor	T11	T12

Neurological level of injury (NLI): **T11**

Complete or incomplete: **I**

Impairment scale: C

Zone of partial preservation

	R	L
Sensory		
Motor		

P4

Onset

Onset	Pin prick(PP) R	Pin prick(PP) L	Light touch (LT) R	Light touch (LT) L
**C2**	2	2	2	2
**C3**	2	2	2	2
**C4**	2	2	2	2
**C5**	2	2	2	2
**C6**	2	2	2	2
**C7**	2	2	2	2
**C8**	2	2	2	2
**T1**	2	2	2	2
**T2**	2	2	2	2
**T3**	2	2	2	2
**T4**	2	2	2	2
**T5**	2	2	2	2
**T6**	2	2	2	2
**T7**	2	2	2	2
**T8**	2	2	2	2
**T9**	1	1	1	1
**T10**	1	1	1	1
**T11**	0	0	0	0
**T12**	0	0	0	0
**L1**	0	0	0	0
**L2**	0	0	0	0
**L3**	0	0	0	0
**L4**	0	0	0	0
**L5**	0	0	0	0
**S1**	0	0	0	0
**S2**	0	0	0	0
**S3**	0	0	0	0
**S4-S5**	0	0	0	0
	**32**	**32**	**32**	**32**

Motor		Right	Left
Elbow Flexor	C5	5	5
Wrist extensor	C6	5	5
Elbow extensor	C7	5	5
Finger flexor	C8	5	5
Finger abductors	T1	5	5
Hip flexors	L2	0	0
Knee exntensors	L3	0	0
Ankle dorsiflexors	L4	0	0
Long toes extensors	L5	0	0
Ankle plantar flexors	S1	0	0
	Total	**25**	**25**

(VAC) Voluntary anal contraction (yes/no): No

Deep Anal Pressure: No

Motor score

UER	**25**	Max (25)
UEL	**25**	(25)
UEMS TOTAL	**50**	(50)
LER	**0**	Max (25)
LEL	**0**	(25)
TOTAL LEMS	**0**	(50)

Sensory Subscores

RLT	**32**	MAX (56)
LLT	**32**	(56)
LT TOTAL	**64**	(112)
RPP	**32**	MAX (56)
LPP	**32**	(56)
PP TOTAL	**64**	(112)

NEUROLOGICAL LEVELS

	R	L
Sensory	T8	T8
Motor	T8	T8

Neurological level of injury (NLI): **T8**

Complete or incomplete: **C**

Impairment scale: A

Zone of partial preservation

	R	L
Sensory	T10	T10
Motor	T10	T10

End

End	Pin prick(PP) R	Pin prick(PP) L	Light touch (LT) R	Light touch (LT) L
**C2**	2	2	2	2
**C3**	2	2	2	2
**C4**	2	2	2	2
**C5**	2	2	2	2
**C6**	2	2	2	2
**C7**	2	2	2	2
**C8**	2	2	2	2
**T1**	2	2	2	2
**T2**	2	2	2	2
**T3**	2	2	2	2
**T4**	2	2	2	2
**T5**	2	2	2	2
**T6**	2	2	2	2
**T7**	2	2	2	2
**T8**	2	2	2	2
**T9**	1	2	1	2
**T10**	1	1	1	1
**T11**	1	1	1	1
**T12**	1	1	1	1
**L1**	1	1	0	0
**L2**	0	1	0	0
**L3**	1	1	0	0
**L4**	1	1	1	1
**L5**	1	1	1	1
**S1**	1	1	1	1
**S2**	0	0	0	0
**S3**	0	0	0	0
**S4-S5**	1	1	0	0
	**40**	**42**	**37**	**38**

Motor		Right	Left
Elbow Flexor	C5	5	5
Wrist extensor	C6	5	5
Elbow extensor	C7	5	5
Finger flexor	C8	5	5
Finger abductors	T1	5	5
Hip flexors	L2	1	1
Knee exntensors	L3	1	1
Ankle dorsiflexors	L4	1	0
Long toes extensors	L5	0	0
Ankle plantar flexors	S1	1	0
	Total	**29**	**27**

(VAC) Voluntary anal contraction (yes/no): Yes

Deep Anal Pressure: Yes

Motor score

UER	**25**	Max (25)
UEL	**25**	(25)
UEMS TOTAL	**50**	(50)
LER	**4**	Max (25)
LEL	**2**	(25)
TOTAL LEMS	**6**	(50)

Sensory Subscores

RLT	**37**	MAX (56)
LLT	**38**	(56)
LT TOTAL	**75**	(112)
RPP	**40**	MAX (56)
LPP	**42**	(56)
PP TOTAL	**82**	(112)

NEUROLOGICAL LEVELS

	R	L
Sensory	T8	T9
Motor	T8	T9

Neurological level of injury (NLI): **T8**

Complete or incomplete: **I**

Impairment scale: C

Zone of partial preservation

	R	L
Sensory		
Motor		

P5

Onset

Onset	Pin prick(PP) R	Pin prick(PP) L	Light touch (LT) R	Light touch (LT) L
**C2**	2	2	2	2
**C3**	2	2	2	2
**C4**	2	2	2	2
**C5**	2	2	2	2
**C6**	2	2	2	2
**C7**	2	2	2	2
**C8**	2	2	2	2
**T1**	2	2	2	2
**T2**	2	2	2	2
**T3**	2	2	2	2
**T4**	2	2	2	2
**T5**	2	2	2	2
**T6**	2	2	2	2
**T7**	2	2	2	2
**T8**	1	1	1	1
**T9**	0	0	0	0
**T10**	0	0	0	0
**T11**	0	0	0	0
**T12**	0	0	0	0
**L1**	0	0	0	0
**L2**	0	0	0	0
**L3**	0	0	0	0
**L4**	0	0	0	0
**L5**	0	0	0	0
**S1**	0	0	0	0
**S2**	0	0	0	0
**S3**	0	0	0	0
**S4-S5**	0	0	0	0
	**29**	**29**	**29**	**29**

Motor		Right	Left
Elbow Flexor	C5	5	5
Wrist extensor	C6	5	5
Elbow extensor	C7	5	5
Finger flexor	C8	5	5
Finger abductors	T1	5	5
Hip flexors	L2	0	0
Knee exntensors	L3	0	0
Ankle dorsiflexors	L4	0	0
Long toes extensors	L5	0	0
Ankle plantar flexors	S1	0	0
	Total	**25**	**25**

(VAC) Voluntary anal contraction (yes/no): No

Deep Anal Pressure: No

Motor score

UER	**25**	Max (25)
UEL	**25**	(25)
UEMS TOTAL	**50**	(50)
LER	**0**	Max (25)
LEL	**0**	(25)
TOTAL LEMS	**0**	(50)

Sensory Subscores

RLT	**29**	MAX (56)
LLT	**29**	(56)
LT TOTAL	**58**	(112)
RPP	**29**	MAX (56)
LPP	**29**	(56)
PP TOTAL	**58**	(112)

NEUROLOGICAL LEVELS

	R	L
Sensory	T7	T7
Motor	T7	T7

Neurological level of injury (NLI): **T7**

Complete or incomplete: **C**

Impairment scale: A

Zone of partial preservation

	R	L
Sensory	T8	T8
Motor	T8	T8

End

End	Pin prick(PP) R	Pin prick(PP) L	Light touch (LT) R	Light touch (LT) L
**C2**	2	2	2	2
**C3**	2	2	2	2
**C4**	2	2	2	2
**C5**	2	2	2	2
**C6**	2	2	2	2
**C7**	2	2	2	2
**C8**	2	2	2	2
**T1**	2	2	2	2
**T2**	2	2	2	2
**T3**	2	2	2	2
**T4**	2	2	2	2
**T5**	2	2	2	2
**T6**	2	2	2	2
**T7**	2	2	2	2
**T8**	2	2	2	2
**T9**	1	1	1	1
**T10**	0	0	1	1
**T11**	0	0	1	1
**T12**	0	0	1	1
**L1**	0	0	0	0
**L2**	0	0	0	0
**L3**	0	0	0	0
**L4**	0	0	0	0
**L5**	0	0	0	0
**S1**	0	0	0	0
**S2**	0	0	0	0
**S3**	0	0	0	0
**S4-S5**	1	1	0	0
	**32**	**32**	**34**	**34**

Motor		Right	Left
Elbow Flexor	C5	5	5
Wrist extensor	C6	5	5
Elbow extensor	C7	5	5
Finger flexor	C8	5	5
Finger abductors	T1	5	5
Hip flexors	L2	1	1
Knee exntensors	L3	1	1
Ankle dorsiflexors	L4	0	0
Long toes extensors	L5	0	0
Ankle plantar flexors	S1	0	0
	Total	**27**	**27**

(VAC) Voluntary anal contraction (yes/no): No

Deep Anal Pressure: Yes

Motor score

UER	**25**	Max (25)
UEL	**25**	(25)
UEMS TOTAL	**50**	(50)
LER	**2**	Max (25)
LEL	**2**	(25)
TOTAL LEMS	**4**	(50)

Sensory Subscores

RLT	**34**	MAX (56)
LLT	**34**	(56)
LT TOTAL	**68**	(112)
RPP	**32**	MAX (56)
LPP	**32**	(56)
PP TOTAL	**64**	(112)

NEUROLOGICAL LEVELS

	R	L
Sensory	T8	T8
Motor	T8	T8

Neurological level of injury (NLI): **T8**

Complete or incomplete: **I**

Impairment scale: C

Zone of partial preservation

	R	L
Sensory		
Motor		

P6

Onset

Onset	Pin prick(PP) R	Pin prick(PP) L	Light touch (LT) R	Light touch (LT) L
**C2**	2	2	2	2
**C3**	2	2	2	2
**C4**	2	2	2	2
**C5**	2	2	2	2
**C6**	2	2	2	2
**C7**	2	2	2	2
**C8**	2	2	2	2
**T1**	2	2	2	2
**T2**	2	2	2	2
**T3**	2	2	2	2
**T4**	2	2	2	2
**T5**	1	1	1	1
**T6**	1	0	1	0
**T7**	0	0	0	0
**T8**	0	0	0	0
**T9**	0	0	0	0
**T10**	0	0	0	0
**T11**	0	0	0	0
**T12**	0	0	0	0
**L1**	0	0	0	0
**L2**	0	0	0	0
**L3**	0	0	0	0
**L4**	0	0	0	0
**L5**	0	0	0	0
**S1**	0	0	0	0
**S2**	0	0	0	0
**S3**	0	0	0	0
**S4-S5**	0	0	0	0
	**24**	**23**	**24**	**23**

Motor		Right	Left
Elbow Flexor	C5	5	5
Wrist extensor	C6	5	5
Elbow extensor	C7	5	5
Finger flexor	C8	5	5
Finger abductors	T1	5	5
Hip flexors	L2	0	0
Knee exntensors	L3	0	0
Ankle dorsiflexors	L4	0	0
Long toes extensors	L5	0	0
Ankle plantar flexors	S1	0	0
	Total	**25**	**25**

(VAC) Voluntary anal contraction (yes/no): No

Deep Anal Pressure: No

Motor score

UER	**25**	Max (25)
UEL	**25**	(25)
UEMS TOTAL	**50**	(50)
LER	**0**	Max (25)
LEL	**0**	(25)
TOTAL LEMS	**0**	(50)

Sensory Subscores

RLT	**24**	MAX (56)
LLT	**23**	(56)
LT TOTAL	**47**	(112)
RPP	**24**	MAX (56)
LPP	**23**	(56)
PP TOTAL	**47**	(112)

NEUROLOGICAL LEVELS

	R	L
Sensory	T4	T4
Motor	T4	T4

Neurological level of injury (NLI): **T4**

Complete or incomplete: **C**

Impairment scale: A

Zone of partial preservation

	R	L
Sensory	T6	T5
Motor	T6	T5

End

End	Pin prick(PP) R	Pin prick(PP) L	Light touch (LT) R	Light touch (LT) L
**C2**	2	2	2	2
**C3**	2	2	2	2
**C4**	2	2	2	2
**C5**	2	2	2	2
**C6**	2	2	2	2
**C7**	2	2	2	2
**C8**	2	2	2	2
**T1**	2	2	2	2
**T2**	2	2	2	2
**T3**	2	2	2	2
**T4**	2	2	2	2
**T5**	2	2	2	2
**T6**	1	1	2	1
**T7**	1	1	1	1
**T8**	1	1	1	1
**T9**	1	1	1	1
**T10**	1	1	1	1
**T11**	1	1	1	1
**T12**	1	1	1	0
**L1**	1	1	0	0
**L2**	0	0	0	0
**L3**	0	0	0	1
**L4**	1	1	1	0
**L5**	1	1	0	0
**S1**	0	0	0	0
**S2**	0	0	0	0
**S3**	1	1	0	0
**S4-S5**	1	1	0	0
	**36**	**36**	**33**	**31**

Motor		Right	Left
Elbow Flexor	C5	5	5
Wrist extensor	C6	5	5
Elbow extensor	C7	5	5
Finger flexor	C8	5	5
Finger abductors	T1	5	5
Hip flexors	L2	1	1
Knee exntensors	L3	1	1
Ankle dorsiflexors	L4	0	0
Long toes extensors	L5	0	0
Ankle plantar flexors	S1	0	0
	Total	**27**	**27**

(VAC) Voluntary anal contraction (yes/no): No

Deep Anal Pressure: Yes

Motor score

UER	**25**	Max (25)
UEL	**25**	(25)
UEMS TOTAL	**50**	(50)
LER	**2**	Max (25)
LEL	**2**	(25)
TOTAL LEMS	**4**	(50)

Sensory Subscores

RLT	**33**	MAX (56)
LLT	**31**	(56)
LT TOTAL	**64**	(112)
RPP	**36**	MAX (56)
LPP	**36**	(56)
PP TOTAL	**72**	(112)

NEUROLOGICAL LEVELS

	R	L
Sensory	T5	T5
Motor	T5	T5

Neurological level of injury (NLI): **T5**

Complete or incomplete: **I**

Impairment scale: C

Zone of partial preservation

	R	L
Sensory		
Motor		

P7

Onset

Onset	Pin prick(PP) R	Pin prick(PP) L	Light touch (LT) R	Light touch (LT) L
**C2**	2	2	2	2
**C3**	2	2	2	2
**C4**	2	2	2	2
**C5**	2	2	2	2
**C6**	2	2	2	2
**C7**	2	2	2	2
**C8**	2	2	2	2
**T1**	2	2	2	2
**T2**	2	2	2	2
**T3**	2	2	2	2
**T4**	2	2	2	2
**T5**	2	2	2	2
**T6**	2	1	2	1
**T7**	1	0	1	0
**T8**	0	0	0	0
**T9**	0	0	0	0
**T10**	0	0	0	0
**T11**	0	0	0	0
**T12**	0	0	0	0
**L1**	0	0	0	0
**L2**	0	0	0	0
**L3**	0	0	0	0
**L4**	0	0	0	0
**L5**	0	0	0	0
**S1**	0	0	0	0
**S2**	0	0	0	0
**S3**	0	0	0	0
**S4-S5**	0	0	0	0
	**27**	**25**	**27**	**25**

Motor		Right	Left
Elbow Flexor	C5	5	5
Wrist extensor	C6	5	5
Elbow extensor	C7	5	5
Finger flexor	C8	5	5
Finger abductors	T1	5	5
Hip flexors	L2	0	0
Knee exntensors	L3	0	0
Ankle dorsiflexors	L4	0	0
Long toes extensors	L5	0	0
Ankle plantar flexors	S1	0	0
	Total	**25**	**25**

(VAC) Voluntary anal contraction (yes/no): No

Deep Anal Pressure: No

Motor score

UER	**25**	Max (25)
UEL	**25**	(25)
UEMS TOTAL	**50**	(50)
LER	**0**	Max (25)
LEL	**0**	(25)
TOTAL LEMS	**0**	(50)

Sensory Subscores

RLT	**27**	MAX (56)
LLT	**25**	(56)
LT TOTAL	**52**	(112)
RPP	**27**	MAX (56)
LPP	**25**	(56)
PP TOTAL	**52**	(112)

NEUROLOGICAL LEVELS

	R	L
Sensory	T6	T5
Motor	T6	T5

Neurological level of injury (NLI): **T5**

Complete or incomplete: **C**

Impairment scale: A

Zone of partial preservation

	R	L
Sensory	T7	T6
Motor	T7	T6

End

End	Pin prick(PP) R	Pin prick(PP) L	Light touch (LT) R	Light touch (LT) L
**C2**	2	2	2	2
**C3**	2	2	2	2
**C4**	2	2	2	2
**C5**	2	2	2	2
**C6**	2	2	2	2
**C7**	2	2	2	2
**C8**	2	2	2	2
**T1**	2	2	2	2
**T2**	2	2	2	2
**T3**	2	2	2	2
**T4**	2	2	2	2
**T5**	2	2	2	2
**T6**	2	2	2	2
**T7**	2	2	2	2
**T8**	2	1	1	1
**T9**	0	0	1	1
**T10**	0	0	0	0
**T11**	0	0	0	0
**T12**	0	0	0	0
**L1**	0	0	0	0
**L2**	0	0	0	0
**L3**	0	0	0	0
**L4**	0	0	0	0
**L5**	0	0	0	0
**S1**	0	0	0	0
**S2**	0	0	0	0
**S3**	0	0	0	0
**S4-S5**	0	0	0	0
	**30**	**29**	**30**	**30**

Motor		Right	Left
Elbow Flexor	C5	5	5
Wrist extensor	C6	5	5
Elbow extensor	C7	5	5
Finger flexor	C8	5	5
Finger abductors	T1	5	5
Hip flexors	L2	1	1
Knee exntensors	L3	0	0
Ankle dorsiflexors	L4	0	0
Long toes extensors	L5	0	0
Ankle plantar flexors	S1	0	0
	Total	**26**	**26**

(VAC) Voluntary anal contraction (yes/no): No

Deep Anal Pressure: No

Motor score

UER	**25**	Max (25)
UEL	**25**	(25)
UEMS TOTAL	**50**	(50)
LER	**1**	Max (25)
LEL	**1**	(25)
TOTAL LEMS	**2**	(50)

Sensory Subscores

RLT	**30**	MAX (56)
LLT	**30**	(56)
LT TOTAL	**60**	(112)
RPP	**30**	MAX (56)
LPP	**29**	(56)
PP TOTAL	**59**	(112)

NEUROLOGICAL LEVELS

	R	L
Sensory	T7	T7
Motor	T7	T7

Neurological level of injury (NLI): **T7**

Complete or incomplete: **C**

Impairment scale: A

Zone of partial preservation

	R	L
Sensory	T9	T9
Motor	L2	L2

P8

Onset

Onset	Pin prick (PP) R	Pin prick (PP) L	Light touch (LT) R	Light touch (LT) L
**C2**	2	2	2	2
**C3**	2	2	2	2
**C4**	2	2	2	2
**C5**	2	2	2	2
**C6**	2	2	2	2
**C7**	2	2	2	2
**C8**	2	2	2	2
**T1**	2	2	2	2
**T2**	2	2	2	2
**T3**	2	2	2	2
**T4**	2	2	2	2
**T5**	2	2	2	2
**T6**	2	2	2	2
**T7**	2	2	2	2
**T8**	2	2	2	2
**T9**	2	2	2	2
**T10**	2	2	2	2
**T11**	2	2	2	2
**T12**	1	1	1	1
**L1**	0	0	0	0
**L2**	0	0	0	0
**L3**	0	0	0	0
**L4**	0	0	0	0
**L5**	0	0	0	0
**S1**	0	0	0	0
**S2**	0	0	0	0
**S3**	0	0	0	0
**S4-S5**	0	0	0	0
	**37**	**37**	**37**	**37**

Motor

		Right	Left
Elbow Flexor	C5	5	5
Wrist extensor	C6	5	5
Elbow extensor	C7	5	5
Finger flexor	C8	5	5
Finger abductors	T1	5	5
Hip flexors	L2	0	0
Knee exntensors	L3	0	0
Ankle dorsiflexors	L4	0	0
Long toes extensors	L5	0	0
Ankle plantar flexors	S1	0	0
	Total	**25**	**25**

(VAC) Voluntary anal contraction (yes/no): No

Deep Anal Pressure: No

Motor score

UER	**25**	Max (25)
UEL	**25**	(25)
UEMS TOTAL	**50**	(50)
LER	**0**	Max (25)
LEL	**0**	(25)
TOTAL LEMS	**0**	(50)

Sensory Subscores

RLT	**37**	MAX (56)
LLT	**37**	(56)
LT TOTAL	**74**	(112)
RPP	**37**	MAX (56)
LPP	**37**	(56)
PP TOTAL	**74**	(112)

NEUROLOGICAL LEVELS

	R	L
Sensory	T11	T11
Motor	T11	T11

Neurological level of injury (NLI): **T11**

Complete or incomplete: **C**

Impairment scale: A

Zone of partial preservation

	R	L
Sensory	T12	T12
Motor	T12	T12

End

End	Pin prick (PP) R	Pin prick (PP) L	Light touch (LT) R	Light touch (LT) L
**C2**	2	2	2	2
**C3**	2	2	2	2
**C4**	2	2	2	2
**C5**	2	2	2	2
**C6**	2	2	2	2
**C7**	2	2	2	2
**C8**	2	2	2	2
**T1**	2	2	2	2
**T2**	2	2	2	2
**T3**	2	2	2	2
**T4**	2	2	2	2
**T5**	2	2	2	2
**T6**	2	2	2	2
**T7**	2	2	2	2
**T8**	2	2	2	2
**T9**	2	2	2	2
**T10**	2	2	2	2
**T11**	2	2	2	2
**T12**	1	1	1	1
**L1**	1	1	1	1
**L2**	1	1	0	1
**L3**	0	1	0	1
**L4**	0	0	0	0
**L5**	0	0	0	0
**S1**	0	1	0	0
**S2**	0	0	0	0
**S3**	1	1	1	1
**S4-S5**	1	1	1	1
	**41**	**43**	**40**	**42**

Motor

		Right	Left
Elbow Flexor	C5	5	5
Wrist extensor	C6	5	5
Elbow extensor	C7	5	5
Finger flexor	C8	5	5
Finger abductors	T1	5	5
Hip flexors	L2	2	2
Knee exntensors	L3	2	2
Ankle dorsiflexors	L4	1	1
Long toes extensors	L5	0	0
Ankle plantar flexors	S1	1	0
	Total	**31**	**30**

(VAC) Voluntary anal contraction (yes/no): Yes

Deep Anal Pressure: Yes

Motor score

UER	**25**	Max (25)
UEL	**25**	(25)
UEMS TOTAL	**50**	(50)
LER	**6**	Max (25)
LEL	**5**	(25)
TOTAL LEMS	**11**	(50)

Sensory Subscores

RLT	**40**	MAX (56)
LLT	**42**	(56)
LT TOTAL	**82**	(112)
RPP	**41**	MAX (56)
LPP	**43**	(56)
PP TOTAL	**84**	(112)

NEUROLOGICAL LEVELS

	R	L
Sensory	T11	T11
Motor	T11	T11

Neurological level of injury (NLI): **T11**

Complete or incomplete: **I**

Impairment scale: C

Zone of partial preservation

	R	L
Sensory		
Motor		
