# Supplementary material for: Training with brain-machine interfaces, visuo-tactile feedback and assisted locomotion improves sensorimotor, visceral, and psychological signs in chronic paraplegic patients
Source: PLoS One. 2018 Nov 29;13(11):e0206464. doi: 10.1371/journal.pone.0206464 (PMC6264837; doi:10.1371/journal.pone.0206464)
Supplement: S2 Fig — (A) MRI cuts of sagittal and (B) axial planes (T2 sequence) at SCI level for patients P2, P3, P4. Myelomalacia (hyperintense signal, 7mm length) is visible for patient P2 at the level of thoracic vertebra T2, and continuity of neural fibers are visible at the lesion level. For patient P3, we observed the spinal cord injury extending between thoracic vertebras T10 and L1 with the presence of remaining fibers (dark gray). T12 axial plane for the same patient reveals injury arachnoiditis (inflammation of spinal meninges) and fiber continuity (better visualization). For patient P4, close to the vertebral body fracture at T8-T9 level, we also observe neural fiber continuity; axial plane reveals dural sac septations at injury level and fiber continuity. (C) Example of 3D segmentation and projection on sagittal and coronal plans for patient P4, confirms spinal cord continuity at lesion level. (PDF) [file pone.0206464.s002.pdf]

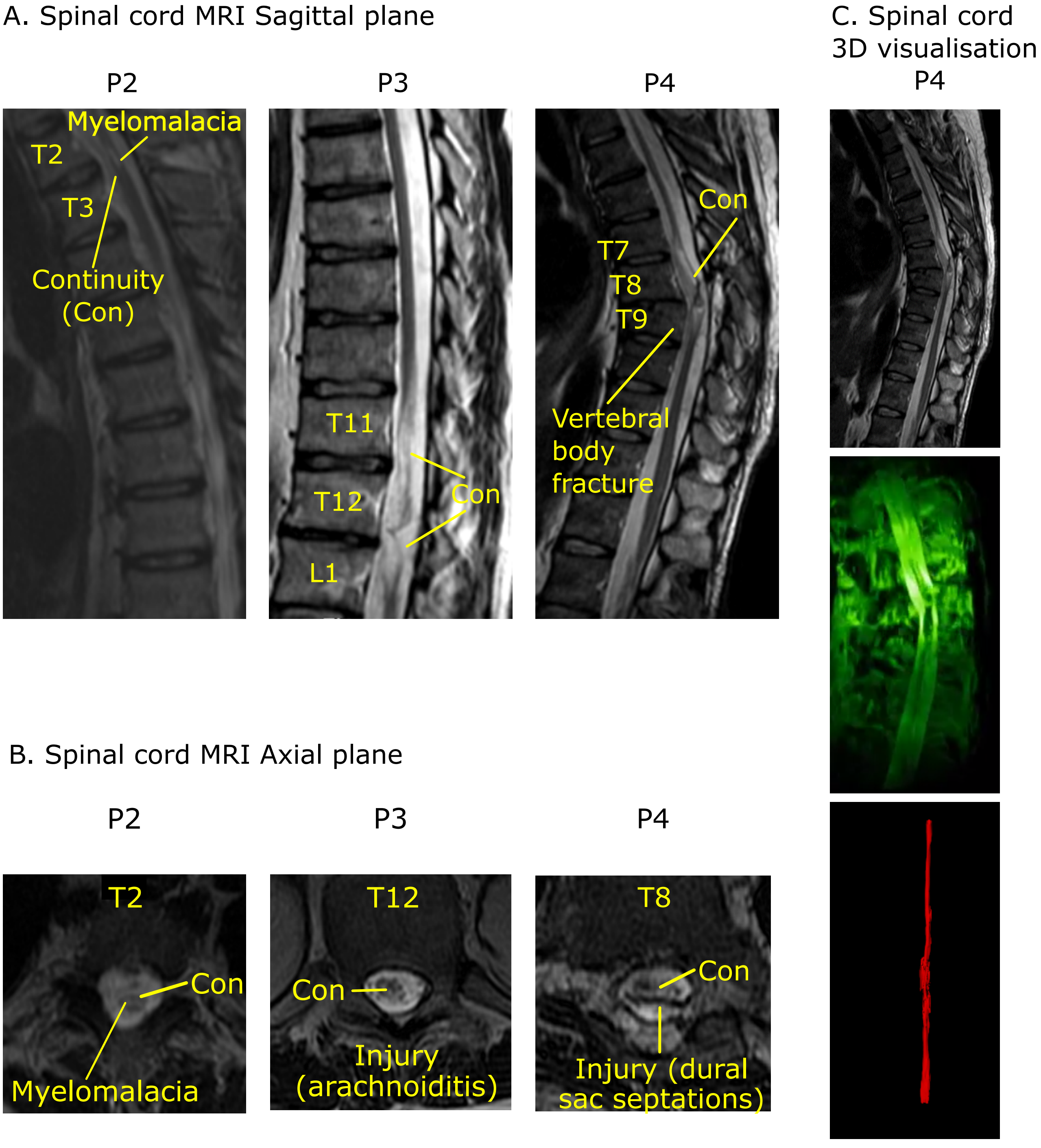

A. Spinal cord MRI Sagittal plane
P2
Myelomalacia
T2
T3
Continuity (Con)
P3
T11
T12
L1
Con
P4
Con
T7
T8
T9
Vertebral body fracture
B. Spinal cord MRI Axial plane
P2
T2
Con
Myelomalacia
P3
T12
Con
Injury (arachnoiditis)
P4
T8
Con
Injury (dural sac septations)
C. Spinal cord 3D visualisation
P4
