# Supplementary material for: Training with brain-machine interfaces, visuo-tactile feedback and assisted locomotion improves sensorimotor, visceral, and psychological signs in chronic paraplegic patients
Source: PLoS One. 2018 Nov 29;13(11):e0206464. doi: 10.1371/journal.pone.0206464 (PMC6264837; doi:10.1371/journal.pone.0206464)
Supplement: S1 Table — (DOCX) [file pone.0206464.s007.docx]

| **Domains** | **Questions** |
| --- | --- |
| **General** **questions** | How would you rate your quality of life? |
|  | How satisfied are you with your health? |
| **Physical** | To what extent do you feel that physical pain prevents you from doing what you need to do? |
|  | How much do you need any medical treatment to function in your daily life? |
|  | Do you have enough energy for everyday life? |
|  | How well are you able to get around? |
|  | How satisfied are you with your sleep? |
|  | How satisfied are you with your ability to perform your daily living activities? |
|  |  |
|  | How satisfied are you with your capacity for work? |
| **Psychological** | How much do you enjoy life? |
|  | To what extent do you feel your life to be meaningful? |
|  | How well are you able to concentrate? |
|  | Are you able to accept your bodily appearance? |
|  | How satisfied are you with yourself? |
|  | How often do you have negative feelings such as blue mood, despair, anxiety, depression? |
| **Social relationships** | How satisfied are you with your personal relationships? |
|  | How satisfied are you with your sex life? |
|  | How satisfied are you with the support you get from your friends? |
| **Environment** | How safe do you feel in your daily life? |
|  | How healthy is your physical environment? |
|  | Have you enough money to meet your needs? |
|  | How available to you is the information that you need in your day-to-day life? |
|  | To what extent do you have the opportunity for leisure activities? |
|  | How satisfied are you with the condition of your living place? |
|  | How satisfied are you with your access to health services? |
|  | How satisfied are you with your transport? |
