# Supplementary material for: Training with brain-machine interfaces, visuo-tactile feedback and assisted locomotion improves sensorimotor, visceral, and psychological signs in chronic paraplegic patients
Source: PLoS One. 2018 Nov 29;13(11):e0206464. doi: 10.1371/journal.pone.0206464 (PMC6264837; doi:10.1371/journal.pone.0206464)
Supplement: S2 Table — The reported principal nerve is the one reported in ASIA assessment (except extensor digitorum longus which is not part of the ASIA assessment). (DOCX) [file pone.0206464.s008.docx]

|  | muscle | nerve name | nerve root range | principle nerve root |
| --- | --- | --- | --- | --- |
| Key muscle | Rectus femoris prox. | femoral nerve | L2-L4 | L2 |
|  | Rectus femoris dist. | femoral nerve | L2-L4 | L3 |
|  | Tibialis anterior | deep fibular nerve | L4-S1 | L4 |
|  | Ext. hallucis longus | deep fibular nerve | L4-S1 | L5 |
|  | Gastrocnemius | tibial nerve | L4-S3 | S1 |
| Non-key muscle | Hip adductors | obturator nerve AND ischiatic nerve | L2-L4 AND L4-S3 | L2 |
|  | Gluteus maximus | inferior gluteal nerve | L5-S2 | L4 |
|  | Gluteus medius | superior gluteal nerve | L4-S1 | L4 |
|  | Medial hamstring | tibial nerve | L4-S3 | L4 |
|  | Lateral hamstring | tibial nerve | L4-S3 | L4 |
|  | Flexor hallucis longus | tibial nerve | L4-S3 | L5 |
|  | Extensor digitorum longus | deep fibular nerve | L4-S1 | L4 |
