# Supplementary material for: Training with brain-machine interfaces, visuo-tactile feedback and assisted locomotion improves sensorimotor, visceral, and psychological signs in chronic paraplegic patients
Source: PLoS One. 2018 Nov 29;13(11):e0206464. doi: 10.1371/journal.pone.0206464 (PMC6264837; doi:10.1371/journal.pone.0206464)
Supplement: S3 Table — (DOCX) [file pone.0206464.s009.docx]

|  | Joint | Flex/Exten | P1 | P2 | P3 | P4 | P5 | P6 | P8 | sum |
| --- | --- | --- | --- | --- | --- | --- | --- | --- | --- | --- |
| Right | Hip | F | 1 | 1 | 1 | 1 | 1 | 1 | 1 | 7 |
|  | Hip | E | 1 | 1 | 1 | 1 | 1 | 1 | 1 | 7 |
|  | Knee | F | 1 | 1 | 1 | 1 | 1 | 1 | 1 | 7 |
|  | Knee | E | 1 | 1 | 1 | 1 | 1 | 1 | 1 | 7 |
|  | Ankle | F | 1 | 1 | 1 | 1 | 0 | 1 | 1 | 6 |
|  | Ankle | E | 1 | 1 | 1 | 1 | 0 | 1 | 1 | 6 |
|  | Hallux | F | 1 | 1 | 1 | 1 | 0 | 0 | 1 | 5 |
|  | Hallux | E | 1 | 1 | 1 | 1 | 0 | 0 | 1 | 5 |
|  | Toes | F | 1 | 1 | 1 | 1 | 0 | 0 | 1 | 5 |
|  | Toes | E | 1 | 1 | 1 | 1 | 0 | 0 | 1 | 5 |
|  |  |  |  |  |  |  |  |  |  |  |
|  |  |  |  |  |  |  |  |  |  |  |
| Left | Hip | F | 1 | 1 | 1 | 1 | 1 | 1 | 1 | 7 |
|  | Hip | E | 1 | 1 | 1 | 1 | 1 | 1 | 1 | 7 |
|  | Knee | F | 1 | 1 | 1 | 1 | 1 | 1 | 1 | 7 |
|  | Knee | E | 1 | 1 | 1 | 1 | 1 | 1 | 1 | 7 |
|  | Ankle | F | 1 | 1 | 1 | 1 | 0 | 1 | 1 | 6 |
|  | Ankle | E | 1 | 1 | 1 | 1 | 0 | 1 | 1 | 6 |
|  | Hallux | F | 1 | 1 | 1 | 1 | 0 | 0 | 1 | 5 |
|  | Hallux | E | 1 | 1 | 1 | 1 | 0 | 0 | 1 | 5 |
|  | Toes | F | 1 | 1 | 1 | 1 | 0 | 0 | 1 | 5 |
|  | Toes | E | 1 | 1 | 1 | 1 | 0 | 0 | 1 | 5 |
